# Hsp70/Bmi1-FoxO1-SOD Signaling Pathway Contributes to the Protective Effect of Sound Conditioning against Acute Acoustic Trauma in a Rat Model

**DOI:** 10.1155/2020/8823785

**Published:** 2020-10-05

**Authors:** Guoxia Zhu, Yongxiang Wu, Yang Qiu, Keyong Tian, Wenjuan Mi, Xinqin Liu, Yuanyuan Chen, Jinwen Jia, Jiasheng Luo, Lianjun Lu, Jianhua Qiu

**Affiliations:** ^1^Department of Otolaryngology-Head and Neck Surgery, Xijing Hospital, Fourth Military Medical University, Xi'an, Shaanxi, China 710032; ^2^Department of Otolaryngology, Head and Neck Surgery, Xi'an People's Hospital/Xi'an Fourth Hospital, Xi'an, Shaanxi, China 710043; ^3^Department of Otolaryngology-Head and Neck Surgery, Chinese PLA General Hospital, Beijing, China 100853; ^4^Department of Otolaryngology-Head and Neck Surgery, General Hospital of Xinjiang Military Region, Urumchi, Xinjiang, China 830011; ^5^Department of Occupational and Environmental Health, Ministry of Education Key Lab of Hazard Assessment and Control in Special Operational Environment and Shaanxi Key Laboratory of Free Radical Biology and Medicine, Fourth Military Medical University, Xi'an, Shaanxi, China 710032; ^6^Department of Otolaryngology, Head and Neck Surgery, The Fifth Affiliated Hospital of Xinjing Medical University, Urumchi, Xinjiang, China 830011; ^7^Department of Otolaryngology-Head and Neck Surgery, Tangdu Hospital, Fourth Military Medical University, Xi'an, China 710038

## Abstract

Sound conditioning (SC) is defined as “toughening” to lower levels of sound over time, which reduces a subsequent noise-induced threshold shift. Although the protective effect of SC in mammals is generally understood, the exact mechanisms involved have not yet been elucidated. To confirm the protective effect of SC against noise exposure (NE) and the stress-related signaling pathway of its rescue, we observed target molecule changes caused by SC of low frequency prior to NE as well as histology analysis in vivo and verified the suggested mechanisms in SGNs in vitro. Further, we investigated the potential role of Hsp70 and Bmi1 in SC by targeting SOD1 and SOD2 which are regulated by the FoxO1 signaling pathway based on mitochondrial function and reactive oxygen species (ROS) levels. Finally, we sought to identify the possible molecular mechanisms associated with the beneficial effects of SC against noise-induced trauma. Data from the rat model were evaluated by western blot, immunofluorescence, and RT-PCR. The results revealed that SC upregulated Hsp70, Bmi1, FoxO1, SOD1, and SOD2 expression in spiral ganglion neurons (SGNs). Moreover, the auditory brainstem responses (ABRs) and electron microscopy revealed that SC could protect against acute acoustic trauma (AAT) based on a significant reduction of hearing impairment and visible reduction in outer hair cell loss as well as ultrastructural changes in OHCs and SGNs. Collectively, these results suggested that the contribution of Bmi1 toward decreased sensitivity to noise-induced trauma following SC was triggered by Hsp70 induction and associated with enhancement of the antioxidant system and decreased mitochondrial superoxide accumulation. This contribution of Bmi1 was achieved by direct targeting of SOD1 and SOD2, which was regulated by FoxO1. Therefore, the Hsp70/Bmi1-FoxO1-SOD signaling pathway might contribute to the protective effect of SC against AAT in a rat model.

## 1. Introduction

Sound conditioning (SC), known as noise-induced “toughening,” is widely defined as acoustic stimulation at a low intensity for an extended period of time prior to an elevated noise exposure (NE), which reduces the permanent threshold shift caused by a high-intensity sound. Clinically, SC has the greatest potential for treating acute acoustic trauma (AAT), as there is currently no other effective therapy. While SC has been shown to have a protective effect in most mammals, the specific mechanisms involved have not yet been explicated. Currently, there are several hypotheses regarding this mechanism: (1) functional reconstruction of OHCs, (2) upregulation of heat shock proteins (Hsp), (3) upregulation of antioxidative enzymes, (4) increases in stress-dependent metabolic activity, (5) alteration of intracellular calcium concentration, (6) increases in blood flow in the cochlea, and (7) changes in lateral efferent functional activity [1, 2]. A previous study suggested that prior to a loud noise, SC (a pure tone of 500 Hz) could enhance the removal of stress-induced free radicals to protect hearing [3]. Then, another study confirmed that SC of the low frequency (a pure tone of 1 kHz) prior to a loud noise upregulated tyrosine hydroxylase in the lateral efferent to protect against acoustic trauma [4]. Furthermore, another work showed that the protective mechanism of hair cells during SC of low frequency (sound conditioning prior to a loud noise) was carried out through an increase in cellular cytoskeleton proteins and through the relief of intracellular calcium overloading caused by NE [5]. Moreover, recent research suggested that the beneficial mechanisms of SC which was prior to a loud noise initiate in the cochlea and eventually reach the central auditory system. This phenomenon might be in part related to an interplay between the calretinin and nitric oxide signaling pathways and increases in the cytosolic calcium buffering capacity induced by SC [6]. In addition, the research by Roy et al. indicated that SC was also protective against two classes of ototoxic drugs (aminoglycosides and cisplatin) [7]. Therefore, it has been widely accepted that preconditioning to sound, especially sound conditioning of low frequency prior to a loud noise (but not sound conditioning after acoustic trauma), is a well-documented strategy to provide protection against AAT and the underlying mechanisms behind the protective effect of SC largely might refer to cochlear tissue.

Interestingly, these data lend further support to the growing body of evidence that specific gene polymorphisms may influence the susceptibility of noise-induced hearing loss, such as Rs3735715 polymorphisms in the GRHL2 gene [8], as well as Rs208679 and rs769217 polymorphisms in the Catalase (CAT) gene [9]. Furthermore, animal research has revealed that homologous animals have different threshold shifts following exposure to noise of the same intensity levels [10]. Pouyatos et al. found that OHCs in a high-frequency area were heavily injured in guinea pigs after the octave band noise (4 kHz, 110 dB, and 8 or 16 kHz at 97 dB) exposure and intervention with an antioxidant inhibitor, while the OHCs from a low-frequency area and the inner hair cells exhibited almost normal [11]. Moreover, exogenous hydrogen peroxide induced a more severe impairment to the OHCs in the high-frequency area than in the other locations [12, 13]. However, antioxidants rescued OHCs in high-frequency areas with a small amount of glutathione, which increased their survival rate, which was generally lower than that of normal guinea pigs [14]. All of the above findings provide evidence that OHCs in high-frequency areas are vulnerable to ROS due to insufficient antioxidation [15]. Thus far, an imbalance in the redox state caused by oxidative stress has been confirmed to play a crucial role in the progress of acoustic trauma [16–18]. Conversely, it is technically and theoretically feasible to enhance the antioxidant system and increase endogenous antioxidant by sound conditioning of low frequency prior to a loud noise, while higher frequencies or even noise might result in hearing loss.

Moreover, regardless of the tissue or cell type, they have their own means of protection against stress-induced acoustic trauma. In most stress incidents such as hypoxia, oxidative stress, temperature shock, and heavy metal poisoning, Hsp70 plays a key role in maintaining protein homeostasis and the correction of protein folding to promote cell survival by directly unfolding misfolded proteins in an ATP-dependent fashion [19]. Conversely, it has been demonstrated that Bmi1, a member of the polycomb group transcription factors, has a significant role in hair cell survival by regulating the redox balance and ROS levels [20, 21]. However, it is still unknown whether both Hsp70 and Bmi1 are genuinely involved in the protective effect of SC against AAT or not.

Therefore, based on the ROS-induced acoustic trauma caused by NE and SC of low frequency prior to a loud noise, the purpose of our study was to investigate the protective effect of SC against AAT and to confirm the stress-related signaling pathway of its rescue. Given the integrity of auditory pathways and the importance of afferent nerves, we specifically focused on both the organ of Corti and the SGNs. Here, we found that SC of low frequency not only protected against AAT based on significant improvements in hearing threshold and an apparent reduction in OHC loss but also improved SGN survival following noise-induced stress response via the increasing amount of mitochondria, regulating mitochondrial function and decreasing ROS levels in rat SGNs, and we first have demonstrated a new theory on the protection of SC against AAT in which upregulation of Hsp70, Bmi1, FoxO1, SOD is involved. Lastly, we suggested that the Hsp70/Bmi1-FoxO1-SOD signaling pathway might contribute to the enhancement of the antioxidant system and a reduction in ROS accumulation for the decreased sensitivity to noise-induced trauma after treatment with SC. Our study was exploratory, and these results from our work could help us easily understand how to protect against AAT by SC and its underlying mechanisms; clinically, these issues will provide a better preventive strategy for AAT.

## 2. Materials and Methods

### 2.1. Animals and Exposure

In this study, 108 healthy adult male Sprague-Dawley rats weighing 200–300 g with a normal Preyer's reflex were provided by the Laboratory Animal Center of the Fourth Military Medical University. The inclusion and exclusion criteria were predetermined that all the animals in this study should have a good hearing without hearing loss caused by tympanitis, drug, noise, genetic problem, and so on. All rats had been raised with sufficient food and water in a tranquil animal cage (the plastic box with railing cover) for 5 days prior to the test (the sound levels were 20-30 dB in each cage in which every four rats were housed).

**Figure 1:**
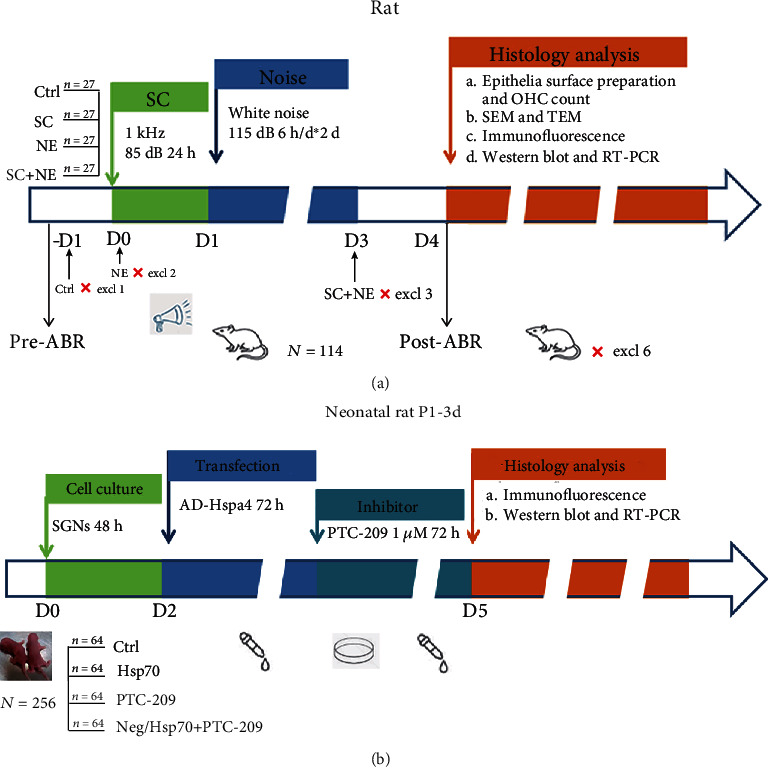
Chronogram of the experiment in vivo (a) and in vitro (b). (a) 114 rats were available in the experiment in vivo, but there were six animals that were excluded based on the exclusion criteria (tympanitis) or died during experiments. Rats were exposed to a pure tone of 1 kHz at 85 dB SPL (SC) for 24 hours; then, some of them and others were exposed to white noise at 115 dB SPL (NE) for 6 hours per day over two consecutive days with anesthesia; they were allowed to rest for 3 hours between SC and NE with anesthesia. Hearing was evaluated with auditory brainstem responses (ABRs) before 1 day prior to exposure and 1 day after exposure. At the end of the study in vivo, cochleae were dissected and processed for histology analysis. (b) The spiral ganglion neuron cells (SGNs) from 256 neonatal rats were harvested and then cultured on culture dishes for 48 h; next, AD-Hspa4 (Hsp70-overexpressing adenovirus) and 1 *μ*M PTC-209 (a small-molecule inhibitor of Bmi1) were, respectively, added to the appropriate cells and incubated for 72 hours at 37°C. At the end of the study in vitro, SGNs were processed for histology analysis.

They were randomly divided into the control group (Ctrl), sound conditioning group (SC), noise exposure group (NE), and sound conditioning plus noise exposure group (SC+NE) by the simple randomization, and the initial number of animals used per group was twenty-seven (Table of random number was used in our study. Each rat had its own number based on its weight which ranged from 1 to 108. Then, every rat was given a random three-digit sequence generated by the random number generator (table of random number) in sequence. Next, these random three-digit sequences which had been assigned to rats were arranged in descending order. Finally, the top 27 rats were matched with Ctrl, the next 27 rats were matched with SC, the last 27 rats were matched with SC+NE, and the rest were matched with NE.). Six animals were excluded based on the exclusion criteria (tympanitis) or died during experiments. One rat in Ctrl, two rats in NE, and three rats in SC+NE were replaced before killing of animals. To minimize animal suffering, an intraperitoneal (i.p.) injection of pentobarbital sodium (30 mg/kg) was used during experiments. The NE group was exposed to white noise at 115 dB SPL for 6 hours per day over two consecutive days with anesthesia to lower stress hormones which might affect the results of the study; in the SC group, all of the animals were exposed to a pure tone of 1 kHz at 85 dB SPL for 24 hours based on Niu and Canlon's study protocol [4]; the Ctrl group was given a sham exposure; the animals in the SC+NE group were exposed to SC before NE and were allowed to rest for 3 hours between SC and NE with anesthesia. The exposure protocol has been described in previous publications from our department [22, 23] (Figure 1(a)). Briefly, exposure was conducted in a ventilated soundproof cabinet where the animals had ad libitum access to food and water except for the rats anesthetized for the noise exposure. A Radio Shack Super Tweeter (Tandy Corp, FT Worth, USA), which was located above the cages, generated a noise (white noise, 115 dB SPL) and a pure tone (1 kHz at 85 dB SPL) that was then amplified by a power amplifier (Yamaha, Japan) and delivered to a loudspeaker. The homogeneity of the sound field was confirmed by a sound level meter (Bruel and Kjaer, China) that was secured within the cabinet.

### 2.2. Auditory Brainstem Response (ABR) Measurement

ABRs to both the click stimuli and the pure tone frequencies in the soundproof chamber on the day before exposure and at approximately 24 hours after the last exposure were available to evaluate hearing alterations in the SD rats in the Ctrl, SC, NE, and SC+NE groups. The ABR experimenter was unaware of the animal's group during ABR measurement. Each animal was gently anesthetized with an intraperitoneal (i.p.) injection of pentobarbital sodium (30 mg/kg) and then was placed on an electric heating plate (37.1–37.5°C) to maintain body temperature. The reference electrode was placed beneath the pinna of the test ear, with the ground electrode placed beneath the apex of the nose, and the active electrode was placed beneath the skin on the top of the head. All of the electrodes were subcutaneously placed at each site within 5 min of the administration of anesthesia. The ABR test started immediately after the needle electrode implantation. Each test ear received the stimulus signal at a repeating rate of 10/s generated through Intelligent Hearing Systems (Bio-Logic Systems, USA), and the stimulus signal was delivered through earphones with a 10 min interval between left and right ears. The signal intensity was decreased gradually by a 5 dB step until the visually discernible ABR waveform disappeared. The lowest sound level that caused this waveform was defined as the “threshold.” Five repetitions of each threshold were presented. The waves were amplified ten times by Intelligent Hearing Systems. The highest sound level was less than 90 dB to avoid drastic acoustic trauma [23, 24].

### 2.3. Scanning Electron Microscopy (SEM) and Transmission Electron Microscopy (TEM)

**Figure 2:**
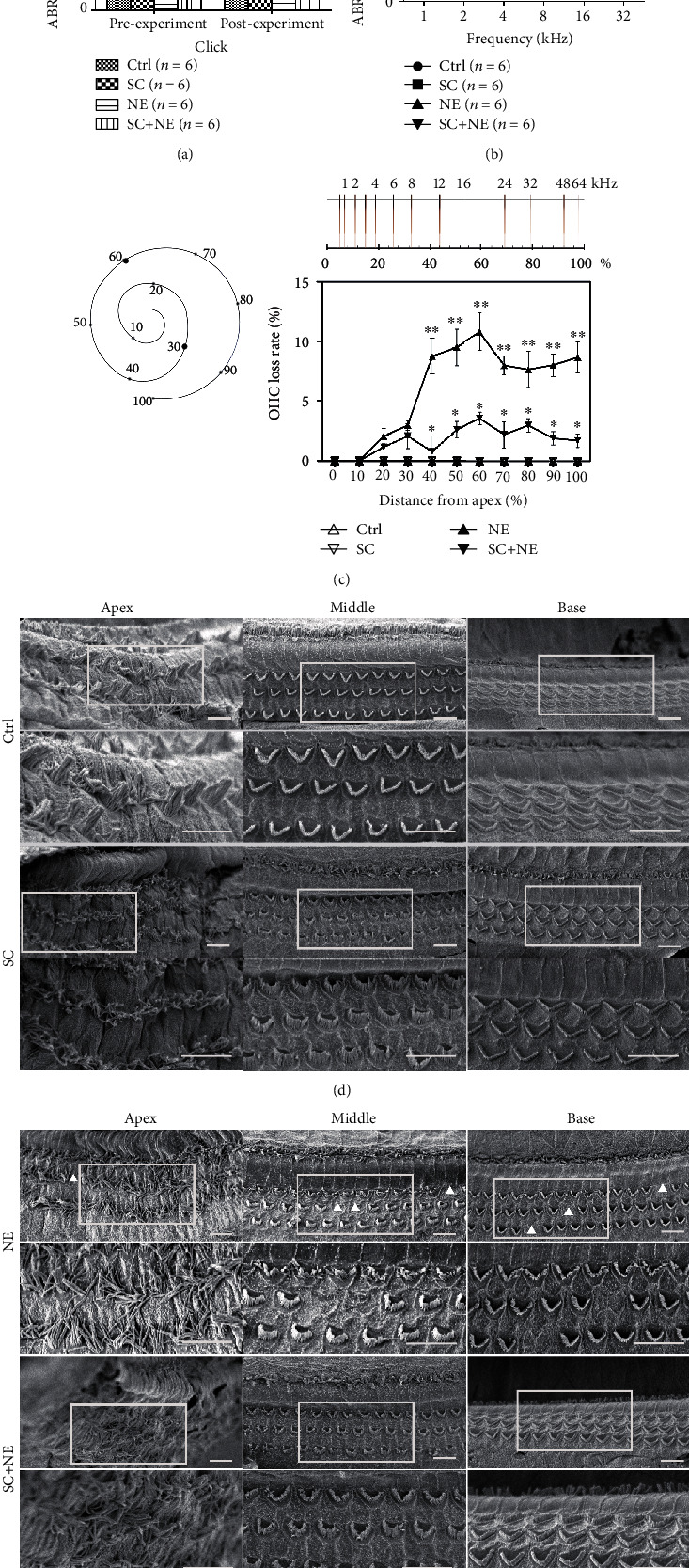
ABR click and tone and basilar membrane and OHC loss. (a) Hearing thresholds of click sound from four groups before and after noise exposure (NE vs. Ctrl: ^∗^*P* < 0.01, SC+NE vs. NE: ^∗∗^*P* < 0.01; mean ± SEM, *n* = 6 animals/group). (b) Hearing thresholds of pure tone from four groups after noise exposure (NE vs. Ctrl: ^∗^*P* < 0.05, SC+NE vs. NE: ^∗∗^*P* < 0.01; mean ± SEM, *n* = 6 animals/group). Ctrl: control group; SC: sound conditioning group which was exposed to a pure tone of 1 kHz at 85 dB SPL for 24 hours; NE: noise exposure group which was exposed to white noise at 115 dB SPL for 6 hours per day over 2 consecutive days; SC+NE: sound conditioning and noise exposure group which was exposed to a pure tone of 1 kHz at 85 dB SPL for 24 hours, and then 3 hours later, followed by white noise at 115 dB SPL for 6 hours per day over 2 consecutive days. Scanning electron micrographs for OHCs in different turns of basilar membrane from the Ctrl and SC groups (d) as well as the NE and SC+NE groups (e). The lower row pictures are the upper row ones at high magnification. White frames show the enlarged areas; triangular arrows indicate OHC loss. Scale bars represent 10 *μ*m. Percentage of OHC loss in different turns from four groups presented in (c) was analyzed with Tukey's multiple comparisons test (Base_NE vs. SC+NE_: ^∗^*P* < 0.05, Middle_NE vs. SC+NE_: ^∗∗^*P* < 0.05; mean ± SD, *n* = 6 pictures from 3 animals/group). Scale is showing frequency and percent distance from the apex for rat cochlea according to Muller (1991). The apex turn of the basilar membrane was considered as the low-frequency area of 0 to 30 percent distance from the apex for the cochlea; the middle turn was the middle-frequency area of 30 to 60 percent distance from the apex, and the base turn was the high-frequency area of 60 to 100 percent distance from the apex.

Cochlear sensory epithelia surface preparation and OHC count in animals from the Ctrl, SC, NE, and SC+NE groups (each group: *n* = 6 from 3 animals) were carried out as described in a previous study from our department. Briefly, following ABR measurement, deeply anesthetized animals from each group were decapitated on the first day after exposure. The cochleae were removed immediately and gently perfused with 2.5% phosphate-buffered glutaraldehyde (pH 7.4) through the open round window and the cochlear apex. The cochleae remained in the same solution overnight. The bony capsule was removed after washing with 0.1 M phosphate-buffered saline (PBS). The spiral ligament and stria vascularis were removed under a dissecting microscope, and the Reissner's membrane was separated. The dissected specimens were rinsed with 0.1 M PBS, then postfixed in 1% osmium tetroxide for 2 hours, and incubated in 2% tannic acid twice for 30 min. The cochleae were dehydrated in a series of graded ethanol solutions and dried in a critical point dryer (Hitachi, Japan). The specimens were fixed on a metal stage, gold-coated in a sputter coater (Ion Sputter, Hitachi, Japan), and observed under SEM (Hitachi, Japan). The experimenter was unaware of the animal's group during the experimentation of OHC count. OHCs were counted by hand using five consecutive images from each slide. The principle of counting is such that if an OHC is not completely contained within the image, the cell was counted only if found at the top or left edge of the image. The missing hair cells and stereocilia were quantified along the entire basilar membrane. The percentage of missing OHCs in each row was calculated and compared among the four groups [22, 23]. In addition, the apex turn of the basilar membrane was considered as the low-frequency area of 0 to 30 percent distance from the apex for the cochlea; the middle turn was the middle-frequency area of 30 to 60 percent distance from the apex, and the base turn was the high-frequency area of 60 to 100 percent distance from the apex. Scale in Figure 2(c) showed frequency and percent distance from the apex for rat cochlea according to Muller's study [25].

Deeply anesthetized animals from each group were perfused transcardially with 0.9% saline followed by a fixative solution of 2.5% glutaraldehyde and 4% paraformaldehyde in 0.1 M PBS (pH 7.4) immediately after the ABR measurements. The cochleae were removed immediately, maintained in the same solution at 4°C for at least 24 hours, and decalcified with 10% EDTA at 23°C for two weeks. Following washing with 0.1 M PBS, the cochleae were divided into small blocks of approximately 1 mm^3^ in size under a dissecting microscope (Olympus, Japan), then fixed with 1% osmic acid for 2 hours, gradually dehydrated in gradient acetone, and embedded in Epon812 for polymerization. Next, the sample blocks were sectioned at a 10 *μ*m thickness and stained with Evans blue to visualize the spiral ganglion. Following visualization, ultrathin 70 nm thick sections were prepared and stained with uranyl acetate and lead citrate following a conventional protocol. Finally, the ultrathin sections were observed under TEM (JEM, Japan) to reveal the ultrastructure of SGNs in the cochlea. The experimenter of electron microscopy was unaware of the animal's group during the experimentation of observation.

### 2.4. Western Blot

Twelve deeply anesthetized rats from each group were sacrificed immediately after the last ABR measurement by decapitation on ice, and the modiolus tissues (or SGN cells *in vitro*) from the same group were harvested, pooled, and stored at −80°C until use. The pooled tissues were lysed in sample buffer containing 1x Tris-EDTA, NaCl (100 mM), 1% Triton X-100, and 1x protease inhibitors. Following mechanical lysis and protein extraction, protein concentration was determined by using a BCA assay kit (Millipore, catalog number BCA1-1KT). Equal amounts (approximately 100 ng/lane) of protein were loaded on 12% sodium dodecyl sulfate-polyacrylamide gels (SDS-PAGE) (Bio-Rad, catalog number 1610173) (120 V for 80 min at room temperature), electrophoresed, and transferred to PVDF membranes (Sigma-Aldrich, catalog number GE10600122) by electroblotting (200 mA for 40 min at 4°C) in 1x transfer buffer (Sigma-Aldrich, catalog number PCG3011). After being blocked in 5% nonfat dry milk and 0.1% Tween-20 in PBS (pH 7.4, 0.01 M) (Sigma-Aldrich, catalog number 9005-64-5) for 1 hour at 25°C, the membranes were incubated overnight at 4°C with different primary antibodies (rabbit anti-FoxO1 polyclonal antibody, 1 : 1000, Cell Signal, catalog number 2880; rabbit anti-Hsp70 polyclonal antibody, 1 : 1000, Abcam, catalog number ab79852; rabbit anti-Bmi1 polyclonal antibody, 1 : 1000, Abcam, catalog number ab38295; rabbit anti-SOD1 polyclonal antibody, 1 : 1000, Proteintech, catalog number 10269-1-AP; or rabbit anti-SOD2 polyclonal antibody, 1 : 1000, GeneTex, catalog number GTX116093). The membranes were then incubated with an HRP-conjugated secondary antibody (goat anti-rabbit antibody, Bioworld, catalog number BS13278) for 1 hour at room temperature. Equal protein loading was confirmed by stripping the blots and reprobing them with a polyclonal rabbit anti-*β*-actin antibody (1 : 1000, GeneTex, catalog number GTX109639) followed by incubation with the same HRP-conjugated secondary antibody. The protein bands were detected by using a chemiluminescence detection technique (FluorChem, Alpha Innotech, USA), and the gray density of the detected bands was analyzed with NIH ImageJ software. The experimenter of western blot was unaware of the animal's group during the experimentation.

### 2.5. Reverse Transcription and Quantitative Real-Time PCR (Quantitative RT-PCR)

This assay was used to detect mRNA expression levels of Hsp70, Bmi1, SOD1, and SOD2 in the rat spiral ganglion cells from the four groups. Total RNA was isolated by TRIzol reagent (Life Technologies Corporation, catalog number 15596026) according to the manufacturer's instructions. Reverse transcription was performed with a First Strand cDNA Synthesis Kit (GeneCopoeia, catalog number AORT-0020) according to the manufacturer's instructions. Quantitative RT-PCR was performed with an All-in-One qPCR Mix (GeneCopoeia, catalog number QP001). Quantitative RT-PCR primers for Hsp70 (RQP051424), Bmi1 (RQP083017), SOD1 (RQP049577), SOD2 (RQP049578), and *β*-actin (RQP051050) were purchased from GeneCopoeia (GeneCopoeia, China). The forward and reverse primers of each PCR set, the sizes of PCR products, GenBank accession numbers, primer IDs, and annealing temperatures are presented in Table 1. RT-PCR was performed for forty cycles with the following parameters: 10 min at 95°C for predenaturation, and in each cycle, 10 s at 95°C for denaturing, 30 s at 60°C for annealing, and 15 s at 72°C for extending (Bio-Rad, USA). All quantitative RT-PCR analyses were conducted with the CFX Manager 3.0 (Bio-Rad, USA). Expression levels of Hsp70 and other genes were normalized to that of *β*-actin by the delta Ct value. The experimenter of PCR was unaware of the animal's group during the experimentation.

### 2.6. Immunofluorescence

Rats from each group were perfused transcardially with freshly prepared 4% paraformaldehyde in 0.1 M PBS (pH 7.4) under deep anesthesia with pentobarbital sodium (60 mg/kg) after ABR measurements. The cochleae were postfixed with the same fixative at 4°C for at least 24 hours, decalcified with 10% EDTA at 23°C for 1 week, dehydrated in 30% sucrose for 24 hours, embedded in OCT glue (SAKURA, catalog number 4583), and sectioned at a 10 *μ*m thickness in the midmodiolus plane on a cryostat (Leica, Germany). The sections were soaked with 0.3% hydrogen peroxide in methanol for 10 min to inactivate endogenous peroxidase and blocked with goat serum (Abcam, catalog number ab7481) at 37°C for 30 min. Then, they were incubated with rabbit anti-Hsp70 polyclonal antibody (1 : 100, Abcam), rabbit anti-Bmi1 polyclonal antibody (1 : 100, Abcam), rabbit anti-FoxO1 monoclonal antibody (1 : 100, Cell Signal), rabbit anti-SOD1 polyclonal antibody (1 : 100, Proteintech) or rabbit anti-SOD2 polyclonal antibody (1 : 100, GeneTex), MitoSOX™ Red mitochondrial superoxide indicator for live-cell imaging (5uM, Invitrogen, catalog number M36008), and mouse anti-tubulin monoclonal antibody (1 : 100, Abcam, catalog number ab7751) at 4°C overnight. Primary antibodies were omitted from the negative controls for these antibodies. Then, sections were incubated with Alexa Fluor 488 Donkey anti-Mouse IgG Highly Cross-Adsorbed Secondary Antibody (1 : 200, Life Technologies, catalog number A-21202) or Alexa Fluor 647 Donkey anti-Mouse IgG Highly Cross-Adsorbed Secondary Antibody (1 : 200, Life Technologies, catalog number A-31571) and Alexa Fluor 594 goat anti-rabbit IgG Highly Cross-Adsorbed Secondary Antibody (1 : 200, Life Technologies, catalog number A-11012) at 37°C for 30 min, followed by staining with DAPI or Hoechst 33258 (1 : 1000, Boster, catalog number AR1176 or catalog number AR1169) at 37°C for 10 min. Three washes in 0.01 M PBS were carried out in the intervals of each step above. Next, the sections were mounted on collagen-coated glass slides and visualized under a fluorescence microscope (Olympus, Japan), while the quantity of target protein expression was analyzed by fluorescence intensity. The experimenter of the fluorescence microscope was unaware of the animal's group during the experimentation of observation.

### 2.7. Cell Culture and Transfection

In this study, 256 healthy neonatal Sprague-Dawley rats (P1-3d) were used for cell culture. Before the experiment starts, cell culture preparation including sterilization and disinfection of cell culture room and operations area and disinfection of work clothes, aseptic mask, sterile gloves, and operating instruments should be strictly implemented, and animals should also be disinfected in time before they are killed quickly. The disinfected animals were sacrificed immediately by decapitation on ice with anesthesia in the disinfected operation room. Then, the modiolus tissues (SGNs) from the killed animals' cochleae were dissected, harvested, and pooled on ice under the stereomicroscope (Leica, Germany) in a super clean bench. After treatment with 0.125% collagenase IV (Thermo Fisher Scientific, catalog number 17104019) and 0.25% trypsinase (Thermo Fisher Scientific, catalog number 25200-056), the spiral ganglion neuron cells (SGNs) from 20 newborn rats per time were cultured on culture dishes in SGN culture medium Dulbecco's modified Eagle's medium with 2% B27 (Thermo Fisher Scientific, catalog number 17504-044), 10 *μ*g/ml brain-derived neurotrophic factor (BDNF, PeproTech, catalog number 450-02), and 1% penicillin-streptomycin solution (100,000 U/l, HyClone, catalog number SV30010) [26, 27]. Then, the SGNs were cultured at 37°C in a humidified incubator (Thermo Scientific, USA) with 5% CO_2_ for 5 days, and the medium was refreshed every 2-3 days. Following a 48-hour incubation, the culture dish of SGNs was divided into four dishes for different interventions, to which 1 *μ*M PTC-209 (a specific inhibitor of Bmi1 with an IC50 value of 0.5 *μ*M, Selleckchem, CAS No. 315704-66-6, catalog number S7372), AD-Hspa4 (2*E* + 10 PFU/ml, 17999-1, GeneChem, China), AD-CON177 (3*E* + 10 PFU/ml, CMV-MCS-3FLAG-SV40-EGFP, GeneChem, China), and enhanced infection solution (GeneChem, China) were added to the appropriate cells and incubated for 72 hours at 37°C. Finally, we observed and disposed of the SGNs from the different procedures [28] (Figure 1(b)). The experimenter of cell culture was unaware of the animal's group during the experimentation of observation.

### 2.8. Statistical Analysis

Experiments were replicated a minimum of three times. The group size (*n*) in vivo was determined by the variability of measurements and the magnitude of the differences between groups. Based on our previous as well as current preliminary studies and sample size calculation (Type I error probability *α*, type II error probability *β*, permissible error *δ*, and standard deviation *S* were used to estimate effect size, when the formula of the sample size required for comparison of means of two samples was performed (*n*_1_ = *n*_2_ = 2[*σ*(*u*_*α*_ + *u*_*β*_)/*u*]^2^ + *u*_*α*_^2^/4).), we determined that six animals per group provide sufficient statistical power (While double-tailed *α* was 0.05 and single-tailed *β* was 0.1 in our study, *u*_*α*/2_ was 1.96 and *u*_*β*_ was 1.282. According to our protocol, standard deviation *S* equaled to *σ*was 2.4 and permissible error *δ* was 5. Finally, the number of animals per group was 5.80 based on the formula of sample size calculation.). Data are presented as means ± SD (standard deviations) or means ± SEM (standard error of mean). The assessment (Q-Q plot) of the normality of data was carried out by SPSS version 17.0 software package (SPSS Inc., Chicago, Ill.). The test (linear regression model) for outliers was conducted on the data by SPSS Data Validation, and no data point was excluded. Two-way analysis of variance was used for comparisons among the different groups that were impacted by two factors, and comparisons with more than two groups were performed with one-way analysis of variance (ANOVA). Further tests included the Student-Newman-Keuls- (SNK-) *q* test for post hoc comparisons or Tukey's multiple comparisons test using GraphPad Prism 5. Statistical analysis of the comparisons between the two groups was accomplished by Student's *t*-test. All tests were two-tailed, and differences were considered to be statistically significant at *P* < 0.05. The experimenters were unaware of the animal's group during experimentation and statistical analysis. Furthermore, the analysis or experimental group assignment was performed by a different person than the experimenter, while all rats were randomly divided into the groups by a simple randomization protocol described in the following. Thus, blinding was achieved in our study.

### 2.9. Compliance with Ethical Standards

All procedures concerning animals in this study were approved by the Institutional Animal Care and Use Committee of the Fourth Military Medical University (Permit number, SYXK 2008-005) and were in compliance with the Guide for the Care and Use of Laboratory Animals.

## 3. Results

### 3.1. SC Protection against Hearing Loss Caused by Acute Noise Exposure

ABR click and tone burst profiles were successfully obtained from rats in each group one day before and after exposure. One-way ANOVA can detect significant changes in hearing function (Figure 2(a)). There was a significant group difference found in postthreshold (*F* = 38.96, *P* < 0.0001) among the four groups. In particular, SNK-*q* tests revealed that there was a significant difference found in the postthreshold between the NE and Ctrl groups (*q* = 13.37, ^∗^*P* < 0.01) and between the NE and SC+NE groups (*q* = 7.56, ^∗∗^*P* < 0.01). However, no significant difference was found between the SC and Ctrl groups (*q* = 0.39, *P* > 0.01).

The ABR hearing threshold for pure tone frequencies was calculated via two-way ANOVA to detect significant changes in the hearing function (Figure 2(b)). Notably, there was a significant group difference (*F* = 302.20, *P* < 0.0001) found among three of the groups (the NE, SC+NE, and Ctrl groups). SNK-*q* tests also showed that there was a significant difference found in the postthreshold between the NE and Ctrl groups (*q* = 76.28, ^∗∗^*P* < 0.001) and between the NE and SC+NE groups (*q* = 15.88, ^∗^*P* < 0.001). However, no significant difference was found between the SC and Ctrl groups (*q* = 8.944, *P* > 0.001). All of these data supported our concept that SC protected against AAT but did not result in hearing impairment on fully mature rats.

### 3.2. Effects of SC and Acute Noise Exposure on Hair Cell Structure

Scanning electron microscopy (SEM) revealed different effects of SC and acute noise exposure on the basilar membrane structure. The results showed that the stereocilia were well organized in “V” or “W” shapes in the Ctrl group. Furthermore, SC resulted in the functional reconstruction of OHCs which included shortening and thickening of the cilia except for misalignment and loosening of the outer hairs in the apex turn of the basilar membrane, while acute noise exposure leads to OHC loss, lodging, fusion, and disappearance of cilia (Figures 2(d) and 2(e)).

Moreover, we analyzed the proportion of OHC loss among the different groups from the base to the apex turns (Figure 2(c)). Tukey's multiple comparisons test revealed that there was a noticeable hair cell loss in both the base and middle turns of the basilar membrane in the NE group compared with the Ctrl group (*F* = 235.5 (column factor: treatment), *P* < 0.0001; *q*_*B*_ = 23.92, ^∗^*P* < 0.05; *q*_*M*_ = 28.68, ^∗^*P* < 0.05), while no significant differences were found between these two groups in the apex turn of the basilar membrane (*q*_*A*_ = 3.70, *P* > 0.05). This finding suggested that acute noise exposure could clearly result in OHC loss in both the base and middle turns but not the apex turn of the basilar membrane. Furthermore, compared with the NE group, less hair cell loss in both the base and middle turns of the basilar membrane was found in the SC+NE group along with shortening and thickening of the cilia, while misalignment and loosening of the outer hairs in the apex turn of the basilar membrane were not improved (*q*_*B*′_ = 17.40, ^∗∗^*P* < 0.05; *q*_*M*′_ = 21.82, ^∗∗^*P* < 0.05). However, this group exhibited no obvious hair cell loss in the apex turn of the basilar membrane with the same reconstruction of OHCs (*q*_*A*′_ = 1.31, *P* > 0.05). This result suggested that SC could protect against OHC loss caused by NE in both the base and middle turns of the basilar membrane on fully mature rats.

### 3.3. Ultrastructural Changes in SGNs Caused by SC and Acute Noise Exposure

**Figure 3:**
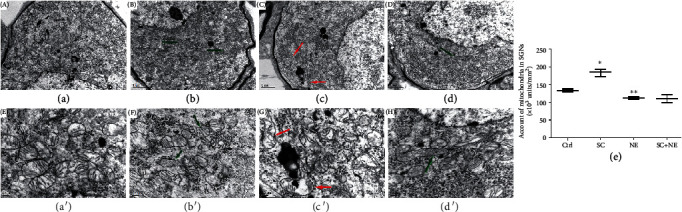
Ultrastructural changes of spiral ganglion neuron. Electron transmission micrographs for SGNs in the control group (a/a′), sound conditioning group (b/b′), noise exposure group (c/c′), and sound conditioning and noise exposure group (d/d′). Green arrows showed that sound conditioning could enhance the electron density in the mitochondrion of spiral ganglion neuron cells and narrow the interspace of the mitochondrion. Red arrows indicated that noise exposure could result in the destruction of the mitochondrion in SGNs, the lower electron density, larger interspace, and the formation of the vacuole in the mitochondrion. Scale bars represent 1 *μ*m and 0.2 *μ*m. The account of a mitochondrion in spiral ganglion neuron cell of SD rat presented in (e) was analyzed with one-way ANOVA (*F* = 55.17; *P* < 0.0001), followed by Newman-Keuls' post hoc test (×10^3^ units/mm^2^, *n* = 6 pictures from 3 animals/group, mean ± SD; Ctrl vs. NE: ^∗∗^*P* < 0.05, SC vs. Ctrl: ^∗^*P* < 0.05).

Transmission electron microscopy (TEM) revealed ultrastructural changes in SGNs caused by SC and acute noise exposure on fully mature rats. SC increased the number of mitochondria in the SGNs, enhanced the electron density of the mitochondria in SGNs, and narrowed the interspace of the mitochondrial matrix. Conversely, NE resulted in the shrinkage of SGNs, decreased the numbers of mitochondria in the SGNs, lowered electron density, and enlarged the interspace between the mitochondrial matrix and the formation of vacuoles in the mitochondria (Figure 3).

We calculated the number of mitochondria in the SGNs of fully mature rats and analyzed their density by the SNK-*q* test. It showed that the densities of mitochondria were significantly different between the NE and Ctrl groups (*q* = 4.78, ^∗∗^*P* < 0.05) and between the SC and Ctrl groups (*q* = 10.86, ^∗^*P* < 0.05) (Figure 3(e)). This result indicated that SC increased the quantity of mitochondria while NE reduced it. But there was no difference between the account of mitochondria in the SC+NE and NE groups shown in Figure 3(e) (*q* = 0.23, *P* > 0.05). It means that NE nullified the protection of mitochondria by SC and SC could largely enhance the density of mitochondria to protect against NE-induced trauma instead of increasing the quantity of mitochondria.

### 3.4. Effects of SC and Acute Noise Exposure on Hsp70, Bmi1, SOD1, SOD2, and FoxO1 Expression Levels in the Rat Spiral Ganglion Cells

**Figure 4:**
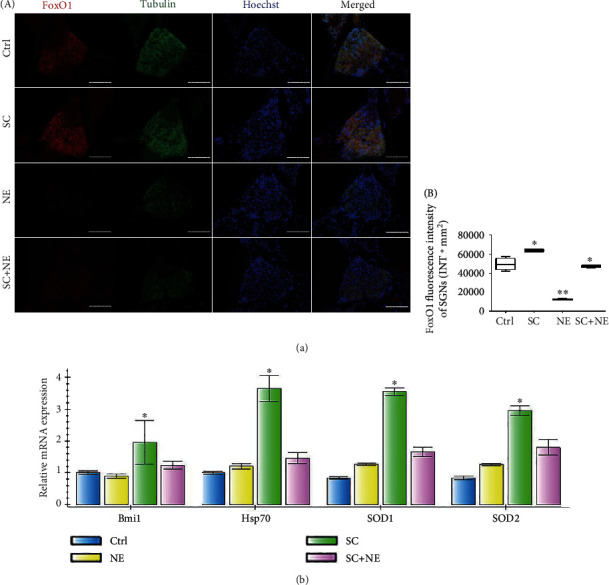
FoxO1 protein and Hsp70, Bmi1, SOD1, and SOD2 mRNA expressions of SGNs after sound conditioning and acute noise exposure. Distributions and expressions of FoxO1 (a) protein were detected with the immune-fluorescence assay in the Ctrl and SC groups as well as the NE and SC+NE groups (A), while the quantity of these protein expressions was analyzed by fluorescence intensity (B). FoxO1 (red) and tubulin (green) as SGN marker were, respectively, labeled with fluorescent secondary antibody, and nuclei (blue) were labeled with Hoechst. Scale bars represent 100 *μ*m (a). Statistical analysis of the results presented in (B) was performed with one-way ANOVA (FoxO1: *F* = 155.3, *P* < 0.0001), followed by Newman-Keuls' post hoc test (^∗^*P* < 0.05, ^∗∗^*P* < 0.05; *n* = 6 pictures from 3 animals/group). Furthermore, values are means ± SD. Relative mRNA expressions of Bmi1, Hsp70, SOD1, and SOD2 were determined by quantitative RT-PCR (b). *β*-Actin RNA level was used as an endogenous control. Values are means ± SD (*n* = 6 animals/group). ^∗^*P* < 0.05 vs. control group.

**Figure 5:**
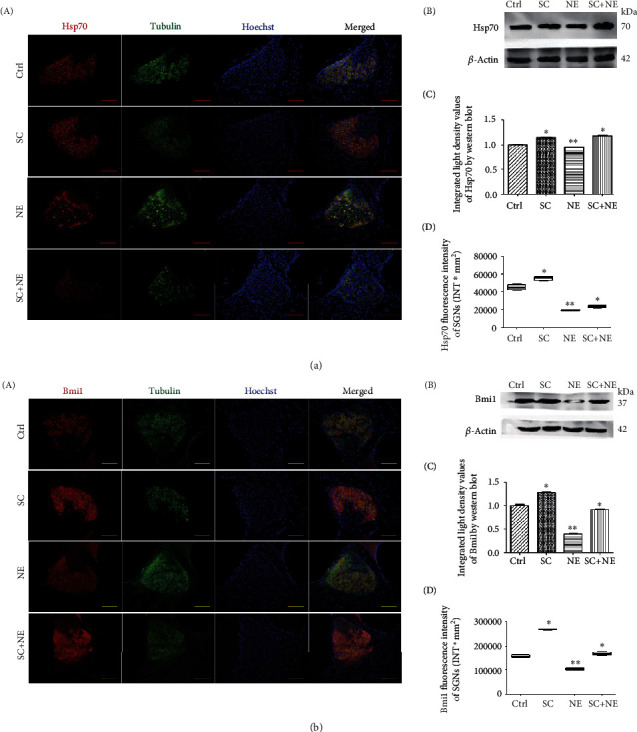
Hsp70 and Bmi1 protein expressions of SGNs after sound conditioning and acute noise exposure. Distributions and expressions of Hsp70 (a) and Bmi1 (b) protein were detected with the immune-fluorescence assay in the Ctrl and SC groups as well as the NE and SC+NE groups (A), while the quantity of these protein expressions was analyzed by fluorescence intensity (D). Hsp70/Bmi1 (red) and tubulin (green) as SGN marker were, respectively, labeled with fluorescent secondary antibody, and nuclei (blue) were labeled with Hoechst. Scale bars represent 100 *μ*m. Protein expressions of these were also assessed with western blot (B), while the quantity of these protein expressions was analyzed by integrated light density (C). *β*-Actin protein was available as an endogenous control. Furthermore, values are means ± SD (*n* = 12 animals/group). ^∗^*P* < 0.05 vs. control group; ^∗∗^*P* < 0.05 vs. control group. Statistical analysis of the results presented in (C) was performed with one-way ANOVA (Hsp70: *F* = 126.5, *P* < 0.0001; Bmi1: *F* = 478.8, *P* < 0.0001), followed by Newman-Keuls' post hoc test (^∗^*P* < 0.05, ^∗∗^*P* < 0.05; *n* = 12 animals/group). Statistical analysis of the results presented in (D) was performed with one-way ANOVA (Hsp70: *F* = 303.4, *P* < 0.0001; Bmi1: *F* = 940.7, *P* < 0.0001), followed by Newman-Keuls' post hoc test (^∗^*P* < 0.05, ^∗∗^*P* < 0.05; *n* = 6 pictures from 3 animals/group). Furthermore, values are means ± SD.

**Figure 6:**
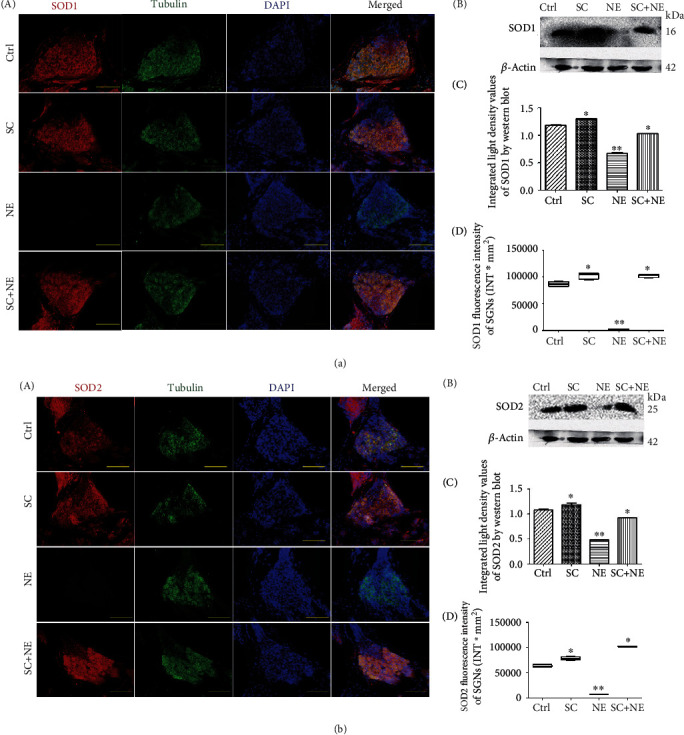
SOD1 and SOD2 protein expressions of SGNs after sound conditioning and acute noise exposure. Distributions and expressions of SOD1 (a) and SOD2 (b) protein were detected with the immune-fluorescence assay in the Ctrl and SC groups as well as the NE and SC+NE groups (A), while the quantity of these protein expressions was analyzed by fluorescence intensity (D). SOD1/SOD2 (red) and tubulin (green) as SGN marker were, respectively, labeled with fluorescent secondary antibody, and nuclei (blue) were labeled with DAPI. Scale bars represent 100 *μ*m. Protein expressions of these were also assessed with western blot (B), while the quantity of these protein expressions was analyzed by integrated light density (C). *β*-Actin protein was available as an endogenous control. Furthermore, values are means ± SD (*n* = 12 animals/group). ^∗^*P* < 0.05 vs. control group; ^∗∗^*P* < 0.05 vs. control group. Statistical analysis of the results presented in (C) was performed with one-way ANOVA (SOD1: *F* = 579.5, *P* < 0.0001; SOD2: *F* = 271.3, *P* < 0.0001), followed by Newman-Keuls' post hoc test (^∗^*P* < 0.05, ^∗∗^*P* < 0.05; *n* = 12 animals/group). Statistical analysis of the results presented in (D) was performed with one-way ANOVA (SOD1: *F* = 1335.0, *P* < 0.0001; SOD2: *F* = 1521, *P* < 0.0001), followed by Newman-Keuls' post hoc test (^∗^*P* < 0.05, ^∗∗^*P* < 0.05; *n* = 6 pictures from 3 animals/group). Furthermore, values are means ± SD.

The IF results revealed that NE lowered Hsp70, Bmi1, FoxO1, SOD1, and SOD2 fluorescence levels in SGNs of a fully mature rat, while SC increased Hsp70, Bmi1, FoxO1, SOD1, and SOD2 fluorescence levels in SGNs (Supplementary Table 1). The WB results of decline at the protein level of Hsp70, Bmi1, SOD1, and SOD2 expressions were found in the NE group, while increased expression of Hsp70, Bmi1, SOD1, and SOD2 expression was found in the SC group (Figures 4(a), 5, and 6 and Supplementary Table 2). In addition, the IF results also revealed that NE lowered Hsp70, Bmi1, SOD1, and SOD2 fluorescence levels in both hair cells and cochlear lateral wall of a fully mature rat, while SC increased Hsp70, Bmi1, SOD1, and SOD2 fluorescence levels in these two regions (Supplementary Figure 3 and 4 and Supplementary Table 1).

Furthermore, the results indicated that SC reversed the downregulation of Hsp70, Bmi1, FoxO1, SOD1, and SOD2 expression caused by NE in SGNs of a fully mature rat. Moreover, RT-PCR exhibited that Hsp70, Bmi1, SOD1, and SOD2 mRNA expression was increased in the SGNs of the SC group compared with that of the Ctrl group (^∗^*P* < 0.05; *n* = 6) (Figure 4(b)).

### 3.5. Overexpression of Hsp70 Significantly Enhanced Bmi1, SOD1, SOD2, and FoxO1 Expression in Rat SGNs and Decreased ROS Accumulation In Vitro

**Figure 7:**
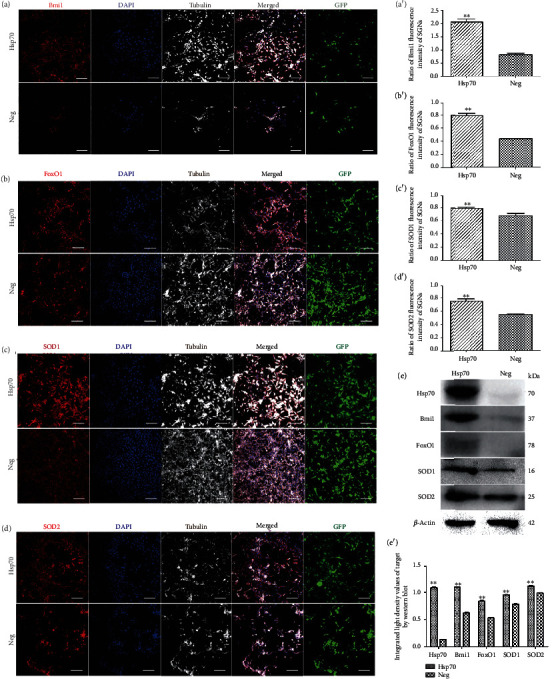
Bmi1, FoxO1, SOD1, and SOD2 expressions of overexpressed HSP70 SGNs. Distributions and expressions of Bmi1, FoxO1, SOD1, and SOD2 protein in SGNs were detected with the immune-fluorescence assay in the Hsp70-overexpressing adenovirus and negative control groups (a–d), while the quantity of these protein expressions was analyzed by the ratio of fluorescence intensity (a′–d′). After successful transfection by Hsp70-overexpressing adenovirus or empty vector adenovirus as GFP (green) shown, Bmi1/FoxO1/SOD1/SOD2 (red) and tubulin (white) as SGN marker were, respectively, labeled with fluorescent secondary antibody and nuclei (blue) were labeled with DAPI. Scale bars represent 100 *μ*m. SGNs expressing a green fluorescent protein (GFP) coded in the same vector as the Hsp70-overexpressing vector was coupled to the GFP sequence, indicating that 66.7% ± 7.8% (*n* = 4 independent cell culture preparations) of the neuronal cells present in the cultures were transfected with Hsp70. Therefore, ratios of Bmi1, FoxO1, SOD1, and SOD2 fluorescence intensity to GFP were calculated. Furthermore, values are means ± SD (*N* = 40 animals from 2 groups). ^∗∗^*P* < 0.05 vs. negative group. Statistical analysis of the results presented in (a′–d′) was performed with Student's *t*-test (^∗∗^*P* < 0.05; *N* = 40 animals from 2 groups). After successful transfection by Hsp70-overexpressing adenovirus or empty vector adenovirus, protein expressions of Hsp70, Bmi1, FoxO1, SOD1, and SOD2 were assessed with western blot (e), while the quantity of these protein expressions was analyzed by integrated light density (e′). *β*-Actin protein was available as an endogenous control. Furthermore, values are means ± SD. ^∗∗^*P* < 0.05 vs. negative group. Statistical analysis of the results was performed with two-way ANOVA (interaction: *F*(4, 20) = 459.8; *P* < 0.0001; row factor: *F*(4, 20) = 493.74; *P* < 0.0001; column factor: *F*(1, 20) = 3400.31; *P* < 0.0001), followed by Student's *t*-test (^∗∗^*P* < 0.05; *n* = 12 animals/group).

Analysis of the neuronal cells on neonatal cultures expressing a green fluorescent protein (GFP) coded in the same vector as the Hsp70-overexpressing vector was coupled to the GFP sequence, which indicated that 66.7% ± 7.8% (*n* = 4 independent cell culture preparations) of the neuronal cells present in the cultures were transfected with Hsp70. After the transfection of SGNs with Hsp70-overexpressing adenovirus *in vitro*, IF results (Figures 7(a)–7(d)/7(a′)–7(d′) and Supplementary Table 1) revealed that ratios of Bmi1, SOD1, SOD2, and FoxO1 fluorescence levels were all increased in the SGNs of neonatal culture compared with the negative control.

**Figure 8:**
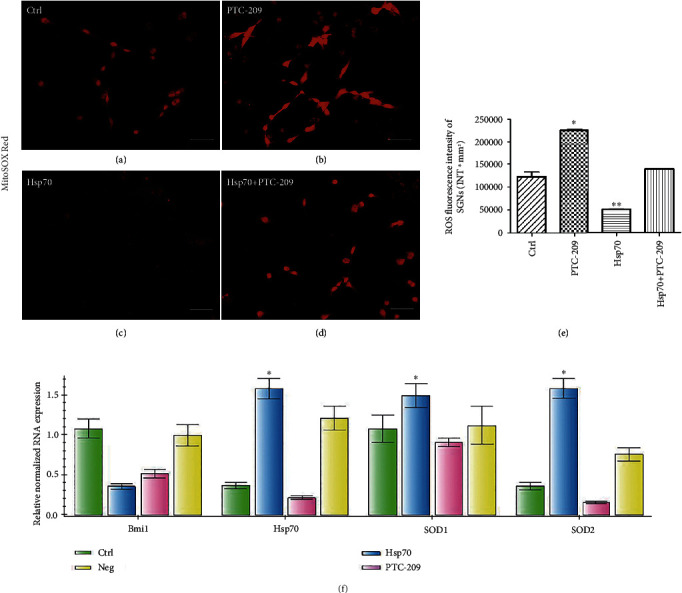
ROS accumulation of different treatments in SGNs and Bmi1, Hsp70, SOD1, and SOD2 mRNA expression of SGNs *in vitro*. ROS accumulation in SGNs was showed by MitoSOX Red and was detected with the immune-fluorescence assay in the control group (a), PTC-209 treated group (b), Hsp70-overexpressing adenovirus group (c), and Hsp70-overexpressing adenovirus followed the PTC-209-treated group (d), while the quantity of ROS accumulation was analyzed by fluorescence intensity (e). Scale bars represent 50 *μ*m. Furthermore, values are means ± SD (*N* = 80). ^∗^*P* < 0.05 vs. control group; ^∗∗^*P* < 0.05 vs. control group. Statistical analysis of the results presented in (e) was performed with one-way ANOVA (*F* = 634.9; *P* < 0.0001), followed by Newman-Keuls' post hoc test (^∗^*P* < 0.05, ^∗∗^*P* < 0.05; *N* = 80 animals from 4 groups). Relative mRNA expressions of Bmi1, Hsp70, SOD1, and SOD2 were determined by quantitative RT-PCR (f). *β*-Actin RNA level was used as an endogenous control. Values are means ± SD (*n* = 12 animals/group). ^∗^*P* < 0.05 vs. control group.

Following transfection of the SGNs with Hsp70 overexpressing the adenovirus on neonatal cultures in vitro, WB results (Figure 7(e)/7(e′) and Supplementary Table 2) also revealed that similar results were found at the protein level of Hsp70, Bmi1, FoxO1, SOD1, and SOD2 expression. There were clearly significant differences between the Hsp70-overexpressing adenovirus and negative control groups (*F* = 3400.31, ^∗∗^*P* < 0.05). Moreover, RT-PCR revealed that Hsp70, SOD1, and SOD2 mRNA expression increases in the SGNs of neonatal culture except for Bmi1 mRNA expression (Figure 8(f) and Supplementary Figure 2).

In addition, there was a significant difference between the Hsp70-overexpressing adenovirus and negative control groups in ROS accumulation labeled by MitoSOX Red (a mitochondrial superoxide indicator for live cells), and the Hsp70-overexpressing adenovirus group (*q* = 31.64, ^∗∗^*P* < 0.05) showed an apparent decrease in ROS accumulation of neonatal culture *in vitro* (Figure 8(a)/8(c)/8(e)).

### 3.6. PTC-209 Decreased the Expression of Bmi1, SOD1, and SOD2 in Rat SGNs and Significantly Increased ROS Accumulation in Rat SGNs In Vitro

**Figure 9:**
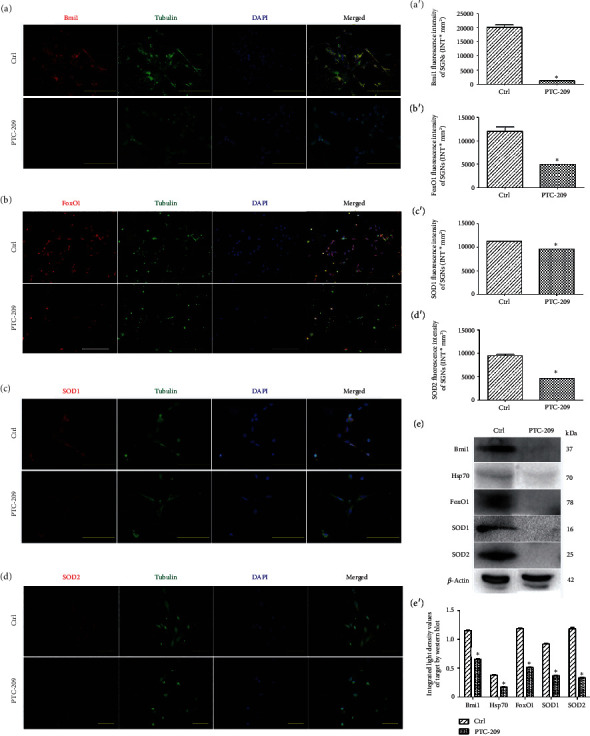
Bmi1, FoxO1, SOD1, and SOD2 expressions in SGNs after PTC-209 treatment. Distributions and expressions of Bmi1, FoxO1, SOD1, and SOD2 protein in SGNs were detected with the immune-fluorescence assay in the control group and the PTC-209 treated groups (a–d), while the quantity of these protein expressions was analyzed by fluorescence intensity (a′–d′). After PTC-209 treatment, Bmi1/FoxO1/SOD1/SOD2 (red) and tubulin (green) as SGN marker were, respectively, labeled with fluorescent secondary antibody and nuclei (blue) were labeled with DAPI. Scale bars represent 100 *μ*m and 50 *μ*m. Furthermore, values are means ± SD (*N* = 40 animals from 2 groups). ^∗^*P* < 0.05 vs. control group. Statistical analysis of the results presented in (a′–d′) was performed with Student's *t*-test (^∗^*P* < 0.05; *N* = 40 animals from 2 groups). After PTC-209 treatment, protein expressions of Hsp70, Bmi1, FoxO1, SOD1, and SOD2 were assessed with western blot (e), while the quantity of these protein expressions was analyzed by integrated light density (e′). *β*-Actin protein was available as an endogenous control. Furthermore, values are means ± SD. ^∗^*P* < 0.05 vs. control group. Statistical analysis of the results was performed with two-way ANOVA (interaction: *F*(4, 20) = 283.78; *P* < 0.0001; row factor: *F*(4, 20) = 1276.15; *P* < 0.0001; column factor: *F*(1, 20) = 7931.17; *P* < 0.0001), followed by Student's *t*-test (^∗^*P* < 0.05; *n* = 12 animals/group).

Following treatment with PTC-209 on neonatal cultures *in vitro*, IF results (Figures 9(a)/9(a′), 9(c)/9(c′), and 9(d)/9(d′) and Supplementary Table 1) revealed that Bmi1, SOD1, and SOD2 fluorescence levels were all decreased in SGNs compared with those in the control group. There were clearly significant differences between the PTC-209 and control groups except for the Hsp70 fluorescence levels (*t* = 0.8032, *P* > 0.05) (Supplementary Figure 1). WB results (Figure 9(e)/9(e′) and Supplementary Table 2) also revealed similar results at the protein level of Bmi1, SOD1, and SOD2 expression with the exception of Hsp70 expression after treatment with PTC-209. There were significant and marked differences between the PTC-209 and the control groups (*F* = 7931.17, ^∗^*P* < 0.05). Moreover, RT-PCR showed that Bmi1, SOD1, and SOD2 mRNA expressions were also clearly decreased in the SGNs on neonatal cultures of the PTC-209 group compared with that of the control group except for Hsp70 mRNA expression (Figure 8(f)).

In addition, there was a significant difference between the PTC-209 and control groups in ROS accumulation of neonatal culture labeled by MitoSOX Red. In the PTC-209 group, ROS accumulation (*q* = 36.17, ^∗^*P* < 0.05) was significantly increased *in vitro*, while a decrease in ROS accumulation of neonatal culture by the Hsp70-overexpressing adenovirus is offset by PTC-209 (Figures 8(a)–8(e)).

### 3.7. FoxO1 Was a Direct Target of Bmi1 in Rat SGNs

After treatment with PTC-209 on neonatal cultures *in vitro*, IF results (Figure 9(b)/9(b′)) revealed that FoxO1 (a transcription factor responsible for the stress response and redox balance) fluorescence levels (*t* = 13.02, ^∗^*P* < 0.05) are lower in the SGNs. WB analysis (Figure 9(e)/9(e′)) also showed that FoxO1 expression (*t* = 50.89, ^∗^*P* < 0.05) is decreased in SGNs on neonatal cultures of the PTC-209 group. There was a significant difference found between the PTC-209 and control groups in FoxO1 expression (*F* = 7931.17, ^∗^*P* < 0.05), which was directly decreased by PTC-209.

## 4. Discussion

**Figure 10:**
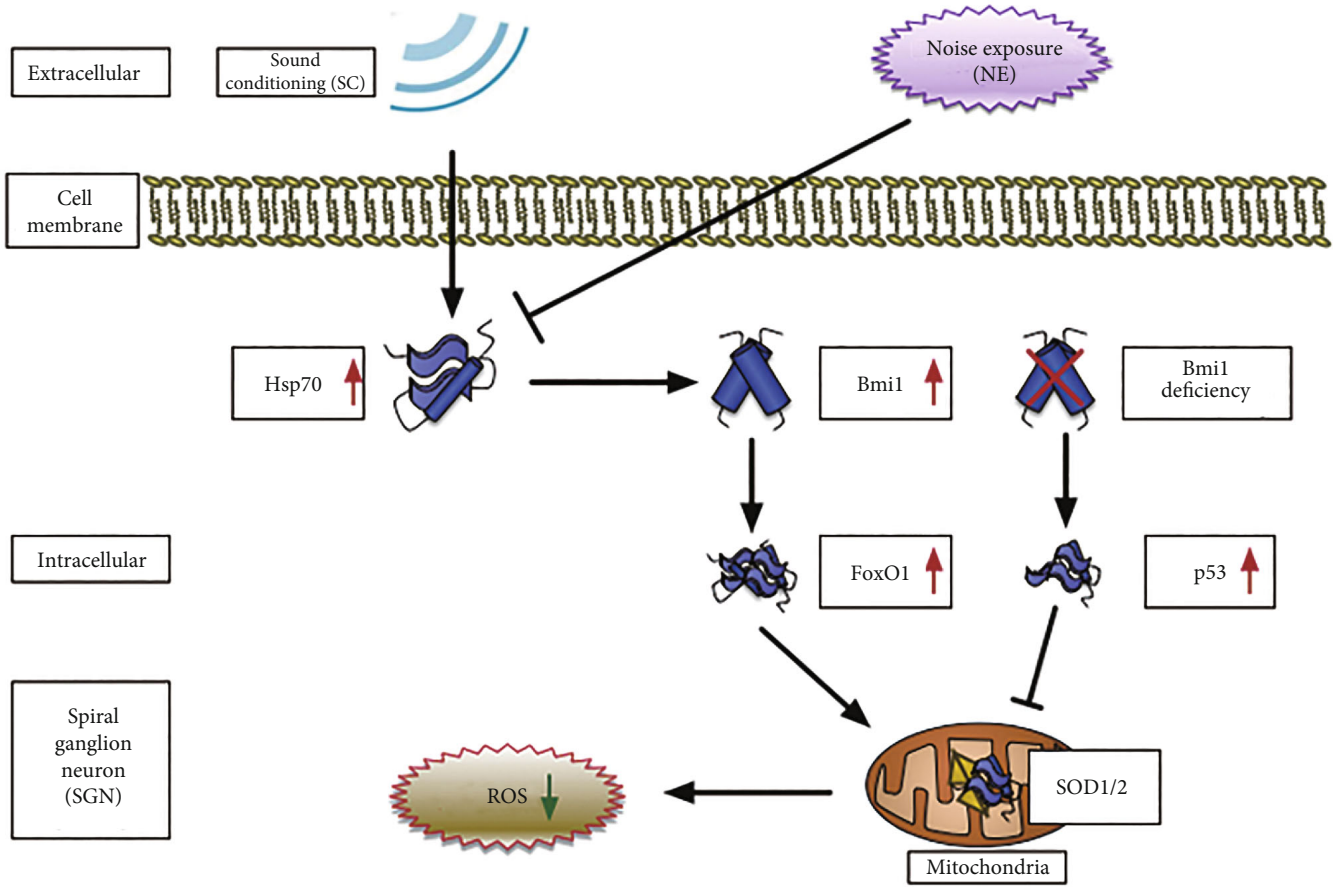
A model for the mechanism of Hsp70-induced Bmi1 upregulation in the protection of sound conditioning against acute acoustic trauma by directly targeting SOD1 and SOD2 regulated by the FoxO1 signaling pathway. Hsp70/Bmi1-FoxO1-SOD1/SOD2 signaling pathway decreases sensitivity in noise-induced trauma following sound conditioning by controlling mitochondrial function and ROS level in rat's SGN. Meanwhile, based on previous studies, other pathway is also involved in Bmi1 deficiency-induced ROS accumulation, such as the following: Bmi1 deficiency directly leads to p53-mediated repression of antioxidant genes, resulting in increased ROS.

Hearing loss is a common sensory disorder all over the world; by far, there are around 466 million patients with hearing disability worldwide. In order to have hearing, HCs are needed for transducing sound vibrations into electrical signaling, and SGNs transmit these electrical signals into the auditory cortex [29–32]. Thus, the majority of the hearing loss is caused by the malfunction or damage of HCs or SGNs. Both HCs or SGNs are vulnerable to be injured by noise, ototoxic drugs, inflammation, biological aging, and genetic defects [16, 33–37]. In recent years, many previous reports used transcription regulation to promote the inner ear stem cells to regenerate the HCs [38–42] and used biomaterials, electrical stimulation, and magnetic regulation to promote the neural stem cells to regenerate the SGNs [43–46] and to promote the maturation of SGNs [47–49]. However, the mammals only have very limited HC and SGN regeneration ability [50–55]; thus, hearing loss is irreversible by far. Thus, to fully understand the detailed protective mechanism of SC in mammals and to effectively protect the HCs and SGNs against various damages are very important. The primary goals of this study were to identify the involvement of Hsp70 and Bmi1 in the protective effect of SC against AAT by targeting SOD1 and SOD2, regulated by the FoxO1 signaling pathway, and to verify the effects of free oxygen radical alteration related to the underlying mechanisms *in vitro* and *in vivo*. In this study, we confirmed that SC prior to a loud noise protected against AAT based on significant improvements in hearing impairment and an apparent reduction in OHC loss (Figure 2) as well as ultrastructural changes in OHCs and SGNs of a fully mature rat (Figure 3). Next, we reported that these functional and ultrastructural changes in rat SGNs were accompanied by significant upregulation of Hsp70, Bmi1, FoxO1, SOD1, and SOD2 protein expression (Figures 5, 6, and 4(a)), which indicated that these proteins were involved in the protective effect of SC against AAT in rats. Furthermore, we used transfection with Hsp70-overexpressing adenovirus and coculture with PTC-209 to verify the Hsp70/Bmi1-FoxO1-SOD signaling pathway and its effect on ROS (mitochondrial superoxide) accumulation of neonatal culture *in vitro* (Figures 7–10). To our knowledge, this is the first report that upregulation of Hsp70 and Bmi1 expression was involved in the protective effect of SC against AAT by targeting SOD1 and SOD2 in rat SGNs, regulated by the FoxO1 signaling pathway.

Previous research lends progressive support to the evidence that oxidative stress, generated in part by glutamate excitotoxicity, impaired mitochondrial function, and GSH depletion resulted in cochlear injury induced by AAT. Therefore, enhancing the cellular oxidative stress defense pathways in the cochlea alleviated noise-induced cochlear injury [56]. Previous studies have further confirmed that AAT is closely related to the degeneration of the antioxidant system in the organs [11–14]. Thus, SC could protect hearing by enhancement of the antioxidant system [1–5, 7, 57–59]. Our results *in vivo* also indicated that SC protected against AAT as it did not result in hearing loss in the studied rats. Furthermore, hair cell loss caused by NE was reduced by SC in both the base and middle turns of the basilar membrane, while the apex is less vulnerable to AAT and SC is of no advantage in that area. Interestingly, SC also increased the quantity of mitochondria, enhanced their function in the SGN cells, and reversed the injury and dysfunction of mitochondria in the SGNs caused by NE. The changes in mitochondria of SGNs were direct proof that the antioxidant system in the organs was enhanced, which contributed to less sensitivity in AAT. Therefore, the findings above in our study fully supported the suggestion that SC could protect against AAT by enhancement of the antioxidant system in the SGNs. Moreover, SC was also reported to be capable of improving the removal of oxidation products by enhancement of the antioxidant system, which protected against AAT [3]. Furthermore, we found that Hsp70, Bmi1, FoxO1, SOD1, and SOD2 were all involved in the SC-induced enhancement of the antioxidant system and reduced ROS accumulation in the SGNs.

It is widely known that the Hsp70 family of heat shock proteins composed of molecular chaperones of approximately 70 kDa in size played a critical role in protein homeostasis. The Hsp70 proteins possess a highly conserved domain structure that is comprised of the following main domains: an ~44 kDa N-terminal nucleotide-binding domain (NBD) which exhibited ATPase activity and was highly conserved; a middle flexible linker region; an ~15 kDa substrate-binding domain (SBD), which interacts with the stretches of the hydrophobic amino acids in the peptides; and an ~10 kDa *α*-helical C-terminal domain that is believed to form a “lid” that closes over the substrate and mediated cochaperone binding [60–63]. Hsp70 proteins played a crucial role in the mediation of correct protein folding and, consequently, in the maintenance of protein homeostasis. Hsp70 directly unfolded misfolded proteins, in adenosine triphosphate- (ATP-) dependent fashion. These proteins also enhanced cell survival following a multitude of stressors, including elevated temperature, hypoxia, oxidative stress, and others. This survival role was reflected in the ability of Hsp70 to buffer the toxicity of denatured and misfolded proteins that accumulated during stress [19]. Many previous studies demonstrated that Hsp induction was a critical stress response in the inner ear that could promote the survival of hair cells exposed to both classes of ototoxic drugs [64–68] and lowered the risks of AAT in mice [69]. In addition, the expression of Hsp70 was increased in the inner ear of mice by exposure to low-frequency noise (LFN), which suggested that Hsp70 played an important part in protecting the inner ears from LFN [70]. Furthermore, strong evidence from recent studies has also shown that SC triggered Hsp70 induction and enhanced its expression *in vivo* [5, 7, 59, 71–73]. According to our study, the RT-PCR, WB, and IF results showed that increased expression of Hsp70 was also involved in the protective effects of SC against AAT in rats. Therefore, we suggested that SC increased Hsp70 expression in the SGNs to enhance cell survival following a noise-induced stress response.

Conversely, evidence from previous studies suggested that Bmi1 improved cell survival by regulating mitochondrial function and ROS levels in the thymocytes and neurons, which had a close bearing on the antioxidants and the removal of oxidation products [3]. Bmi1, a member of the polycomb protein family, could bind to the Runx1/CBF*β* transcription factor complex to silence target genes in a PRC2-independent manner [74]. Abdouh et al. confirmed that other than in stem cells and rapidly dividing cells, Bmi1 was expressed in terminally differentiated cells such as neurons and increased in Bmi1 expression in cortical neurons which activated antioxidant genes to regulate ROS generation and protected against DNA damage-induced cell apoptosis as well as mitochondrial injury [75]. In neurons, Bmi1 also suppressed p53-induced cell apoptosis by regulating antioxidation [76]. Furthermore, a previous report also showed that Bmi1-deficient thymocytes impaired mitochondrial function, which led to a marked increase in intracellular ROS levels and a subsequent engagement of the DDR pathway [77]. Rizo et al. found that a reduced ability of self-renewal was associated with enhanced apoptosis in Bmi1- CD34(+) stem cells, which coincided with increased levels of intracellular ROS [78]. Furthermore, overexpression of Bmi1 in vivo protected human embryonic stem cells (HSCs) from ROS damage and extended the lifespan of HSCs [79], whereas Bmi1 transduction in vitro reduced irradiation-induced ROS levels by suppressing the oxidase genes and increased repair of the DNA damage in human keratinocytes [80]. Furthermore, in a recent study by Chen et al., Bmi1 was demonstrated to have an important role in hair cell survival via controlling the redox balance and ROS levels [20]. Our study in vivo and in vitro produced similar results to Chen et al. However, the current study also found that these functional and ultrastructural changes of mitochondria were accompanied by significant upregulation of Hsp70 and Bmi1 expression induced by SC and that overexpression of Hsp70 significantly enhanced Bmi1 expression in rat SGNs (Hsp70 might stabilize Bmi1 at the protein level rather than increase it at the transcriptional level) and clearly decreased mitochondrial superoxide accumulation, while Bmi1 inhibitor attenuated the effect of Hsp70 transfection. This finding suggested that SC induce increased Bmi1 expression in the SGNs, which was triggered by Hsp70 induction to improve cell survival by regulating mitochondrial function and mitochondrial superoxide levels.

It is widely accepted that several pathways are involved in Bmi1 deficiency-induced ROS accumulation, including the following: (1) Bmi1 deficiency leads to mitochondrial dysfunction resulting in a rise of intracellular ROS and subsequent engagement of the DDR pathway, in which Chk2 and p53 were activated [77]; (2) Bmi1 deficiency directly leads to p53-mediated repression of the antioxidant genes, resulting in increased ROS levels [75]. However, how to control mitochondrial function and ROS level in SGNs by Bmi1 upregulation and the mechanism by which SC exerted a protective effect against AAT have remained unknown. To fix this problem, we further investigated FoxO1 (a transcription factor responsible for the stress response and redox balance) [81], SOD1 (an antioxidant gene whose protein is found in the cytoplasm), and SOD2 (an antioxidant gene whose protein is found in the mitochondrial ridge) expressions in SGNs *in vivo* and *in vitro* by transfection with Hsp70-overexpressing adenovirus and coculture with PTC-209. Ultimately, we found that functional and ultrastructural changes in the mitochondria were still associated with a significant upregulation of FoxO1, SOD1, and SOD2 expression induced by SC and we also confirmed that PTC-209 significantly decreased FoxO1, SOD1, and SOD2 expressions in the rat SGNs and increased mitochondrial superoxide accumulation, while overexpression of Hsp70 significantly reversed these expressions in the rat SGNs. We suggested that changes in SOD1/SOD2 expression could be involved in the protective effects of SC and a consequence of cell protection. Therefore, our results indicated that the Hsp70/Bmi1-FoxO1-SOD signaling pathway was involved in the protective effect of SC against AAT and regulated mitochondrial function and the mitochondrial superoxide levels in rat SGNs (Figure 10), which coincided with the regulation of oxidative stress and mitochondrial dysfunction by FoxO1 in human QBC939 cells from a recent study [82]. Meanwhile, the involvement of the Hsp70/Bmi1-FoxO1-SOD signaling pathway in other regions of cochlear tissue (such as hair cells and cochlear lateral wall) behind the protective effect of SC has also been conformed further by our immunolabeling data. However, in view of the complexity of regulating Bim1 expression in SGNs, we still believe that there might be additional signaling pathways that regulate mitochondrial function and ROS levels in rat SGNs. Therefore, more research on this issue should be performed. In addition, the overexpression experiments on cultured SGN were performed on neonatal cultures whereas our noise exposure/preconditioning experiments were performed on fully mature rats. Although immature SGNs differ in many ways from mature ones (expression of ion channels, etc.) [83], there is no significant difference between the changes of a target molecule in vivo and in vitro based on our current research data.

Thus, we found that SC of low frequency prior to a loud noise not only protected against AAT based on significant improvements in hearing impairment and an apparent reduction in OHC loss but also improved SGN survival following a noise-induced stress response via controlling mitochondrial function and ROS levels in rat SGNs, and we are the first to have demonstrated a new theory on the protection of SC against AAT in which the Hsp70/Bmi1-FoxO1-SOD signaling pathway was involved. Lastly, we suggested that the enhancement of the antioxidant system and a reduction in ROS accumulation might be the main reasons for decreased sensitivity to noise-induced trauma following treatment with SC. The results from our work help us to understand the protective capacity of SC against AAT and the underlying mechanisms involved.

## 5. Conclusions and Future Directions

In summary, the in vitro and in vivo data presented here provide evidence that (i) SC improved SGN survival following noise-induced stress response via controlling mitochondrial function and ROS levels, (ii) Hsp70/Bmi1-FoxO1-SOD signaling pathway was involved in the protection of SC against AAT, and (iii) enhancement of the antioxidant system and a reduction in ROS accumulation might be the main reasons for the decreased sensitivity to AAT following treatment with SC in the rats. Clinically, these issues could provide a better preventive strategy for AAT.

### 5.1. Limitations of Our Work

Due to technical issues, the harvested tissues in western blot included other supporting cells in addition to SGN so that the data of western blot does not coincide with that of immunofluorescence in vivo (especially for Hsp70). This issue might be resolved by tissue purification technology. The roles of Hsp70 and Bmi1 have been demonstrated in vitro but not fully in vivo. The role of FoxO1 in the upregulation of SOD should be demonstrated further. Furthermore, so far, it is still unclear how the FoxO1 signaling pathway activates antioxidant genes to control mitochondrial function and ROS levels. Finally, whether the SC stimulus of other higher frequencies or even noise might protect against hearing loss more effectively or not should be considered to study further. Therefore, more research on these issues should be performed in our next study.

## Figures and Tables

**Table 1:** Primers used for quantitative RT-PCR.

Gene	Lot No.	Primer ID	Amplicon size	GenBank accession number	Annealing temperature
Hsp70	RQP051424	Rn-QRP-10166	133	NM_031971.2	60°C
Bmi1	RQP083017	Rn-QRP-10984	119	NM_001107368.2	60°C
SOD1	RQP049577	Rn-QRP-10681	128	NM_017050.1	60°C
SOD2	RQP049578	Rn-QRP-10168	91	NM_017051.2	60°C
*β*-Actin	RQP051050	Rn-QRP-10046	98	NM_031144.2	60°C

## Data Availability

The data used to support the findings of this study were supplied by Jianhua Qiu under license and so cannot be made freely available. Requests for access to these data should be made to Jianhua Qiu (qiujh@fmmu.edu.cn).

## Supplementary Materials

Supplementary Materials Supplementary 1. Supplementary Figure 1: Hsp70 expression in SGNs after PTC-209 treatment distribution and expression of Hsp70 protein in SGNs was detected with immunofluorescence assay in the control group and PTC-209 treated groups (A), while the quantity of Hsp70 protein expression was analyzed by fluorescence intensity (B). After PTC-209 treatment, Hsp70 (red) and tubulin (green) as SGN marker were, respectively, labeled with fluorescent secondary antibody and nuclei (blue) were labeled with DAPI. Scale bars represent 50 *μ*m. Furthermore, values are means ± SD (*N* = 40 animals from 2 groups). *P* > 0.05 vs. control group. Statistical analysis of the results presented in (B) was performed with Student's *t*-test (*P* > 0.05; *N* = 40 animals from 2 groups). Supplementary 2. Supplementary Figure 2: Hsp70, SOD1, SOD2, and Bmi1 mRNA expression of SGNs *in vitro*. Relative mRNA expressions of Hsp70, SOD1, SOD2, and Bmi1 were determined by quantitative RT-PCR. *β*-Actin RNA level was used as an endogenous control. Values are means ± SD (*n* = 12 animals/group). ^∗^*P* < 0.05 Hsp70 vs. control group. Supplementary 3. Supplementary Figure 3: Hsp70 and Bmi1 protein expressions in hair cells and cochlear lateral wall after sound conditioning and acute noise exposure. Distributions and expressions of Hsp70 (A) and Bmi1 (B) protein were detected with immunofluorescence assay in the Ctrl and SC groups as well as the NE and SC+NE groups (a), while the quantity of these protein expressions in both hair cells (b) and cochlear lateral wall (c) was analyzed by fluorescence intensity. Hsp70/Bmi1 (red) was labeled with fluorescent secondary antibody, and nuclei (blue) were labeled with Hoechst. Scale bars represent 100 *μ*m. Furthermore, values are means ± SD. Statistical analysis of the results presented in (b) (Hsp70: *F* = 80.74, *P* < 0.0001; Bmi1: *F* = 86.64, *P* < 0.0001) and (c) (Hsp70: *F* = 684.6, *P* < 0.0001; Bmi1: *F* = 338.2, *P* < 0.0001) was performed with one-way ANOVA, followed by Newman-Keuls' post hoc test (^∗^*P* < 0.05, ^∗∗^*P* < 0.05; *n* = 6 pictures from 3 animals/group). Supplementary 4. Supplementary Figure 4: SOD1 and SOD2 protein expressions in hair cells and cochlear lateral wall after sound conditioning and acute noise exposure. Distributions and expressions of SOD1 (A) and SOD2 (B) protein were detected with immunofluorescence assay in the Ctrl and SC groups as well as the NE and SC+NE groups (a), while the quantity of these protein expressions in both hair cells (b) and cochlear lateral wall (c) was analyzed by fluorescence intensity. SOD1/SOD2 (red) was labeled with fluorescent secondary antibody, and nuclei (blue) were labeled with Hoechst. Scale bars represent 100 *μ*m. Furthermore, values are means ± SD. Statistical analysis of the results presented in (b) (SOD1: *F* = 233.0, *P* < 0.0001; SOD2: *F* = 79.93, *P* < 0.0001) and (c) (SOD1: *F* = 143.0, *P* < 0.0001; SOD2: *F* = 199.8, *P* < 0.0001) was performed with one-way ANOVA, followed by Newman-Keuls' post hoc test (^∗^*P* < 0.05, ^∗∗^*P* < 0.05; *n* = 6 pictures from 3 animals/group). Supplementary 5. Supplementary Table 1: statistical results of immunofluorescence of target protein expression in vivo and in vitro (Student-Newman-Keuls- (SNK-) *q* test for post hoc comparisons (NE or SC vs. Ctrl) ^∗∗^*P* < 0.05, ^∗^*P* < 0.05; Student's *t*-test for the comparisons between the two groups (Hsp70 vs. Neg or PTC-209 vs. Ctrl) ^∗∗^*P* < 0.05, ^∗^*P* < 0.05). Supplementary 6. Supplementary Table 2: statistical results of western blot of target protein expression in vivo and in vitro (Student-Newman-Keuls- (SNK-) *q* test for post hoc comparisons (NE or SC vs. Ctrl) ^∗∗^*P* < 0.05, ^∗^*P* < 0.05; Student's *t*-test for the comparisons between the two groups (Hsp70 vs. Neg or PTC-209 vs. Ctrl) ^∗∗^*P* < 0.05, ^∗^*P* < 0.05).
