# Supplementary material for: Hsp70/Bmi1-FoxO1-SOD Signaling Pathway Contributes to the Protective Effect of Sound Conditioning against Acute Acoustic Trauma in a Rat Model
Source: Neural Plast. 2020 Oct 5;2020:8823785. doi: 10.1155/2020/8823785 (PMC7556106; doi:10.1155/2020/8823785)
Supplement: Supplementary Materials — Supplementary 1. Supplementary Figure 1: Hsp70 expression in SGNs after PTC-209 treatment distribution and expression of Hsp70 protein in SGNs was detected with immunofluorescence assay in the control group and PTC-209 treated groups (A), while the quantity of Hsp70 protein expression was analyzed by fluorescence intensity (B). After PTC-209 treatment, Hsp70 (red) and tubulin (green) as SGN marker were, respectively, labeled with fluorescent secondary antibody and nuclei (blue) were labeled with DAPI. Scale bars represent 50 μm. Furthermore, values are means ± SD (N = 40 animals from 2 groups). P > 0.05 vs. control group. Statistical analysis of the results presented in (B) was performed with Student's t-test (P > 0.05; N = 40 animals from 2 groups). Supplementary 2. Supplementary Figure 2: Hsp70, SOD1, SOD2, and Bmi1 mRNA expression of SGNs in vitro. Relative mRNA expressions of Hsp70, SOD1, SOD2, and Bmi1 were determined by quantitative RT-PCR. β-Actin RNA level was used as an endogenous control. Values are means ± SD (n = 12 animals/group). ∗P < 0.05 Hsp70 vs. control group. Supplementary 3. Supplementary Figure 3: Hsp70 and Bmi1 protein expressions in hair cells and cochlear lateral wall after sound conditioning and acute noise exposure. Distributions and expressions of Hsp70 (A) and Bmi1 (B) protein were detected with immunofluorescence assay in the Ctrl and SC groups as well as the NE and SC+NE groups (a), while the quantity of these protein expressions in both hair cells (b) and cochlear lateral wall (c) was analyzed by fluorescence intensity. Hsp70/Bmi1 (red) was labeled with fluorescent secondary antibody, and nuclei (blue) were labeled with Hoechst. Scale bars represent 100 μm. Furthermore, values are means ± SD. Statistical analysis of the results presented in (b) (Hsp70: F = 80.74, P < 0.0001; Bmi1: F = 86.64, P < 0.0001) and (c) (Hsp70: F = 684.6, P < 0.0001; Bmi1: F = 338.2, P < 0.0001) was performed with one-way ANOVA, followed by Newman-Keuls' post h [file 8823785.f1.docx]

**Supplement file**


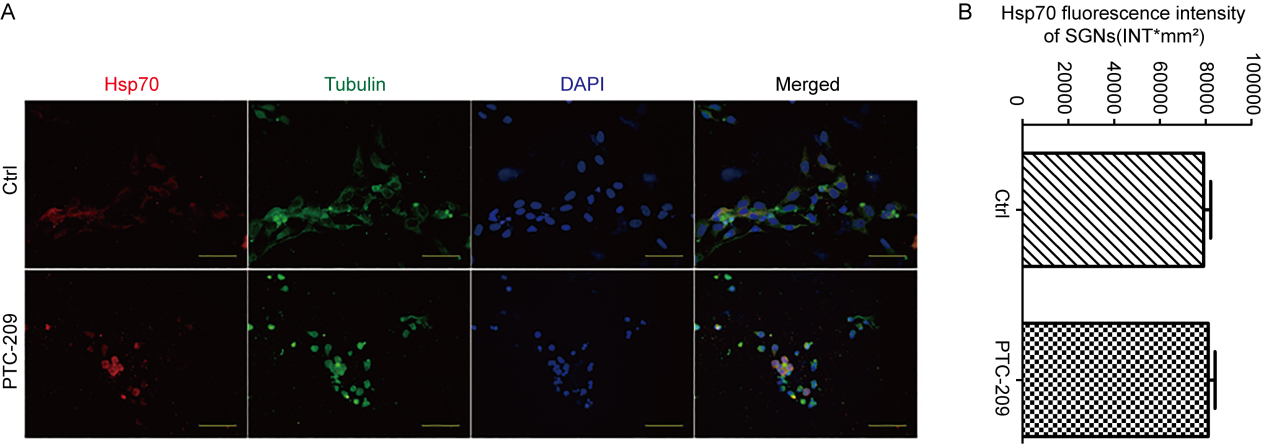


*Supplementary 1.* Supplementary Figure 1**: Hsp70 Expression in SGNs after PTC-209 Treatment** Distribution and expression of Hsp70 protein in SGNs was detected with immunofluorescence assay in Control group and PTC-209 treated groups **(A)**, while quantity of Hsp70 Protein expression was analyzed by fluorescence intensity **(B)**. After PTC-209 treatment, Hsp70 (red) and Tubulin (green) as SGN marker were, respectively, labeled with fluorescent secondary antibody and nuclei (blue) were labeled with DAPI. Scale bars represent 50µm. Furthermore, values are Means ± SD (N=40 animals from 2 groups). *P* > 0.05 vs. control group. Statistical analysis of the results presented in (**B**) was performed with Student’s *t*-test (*P*>0.05; N=40 animals from 2 groups).


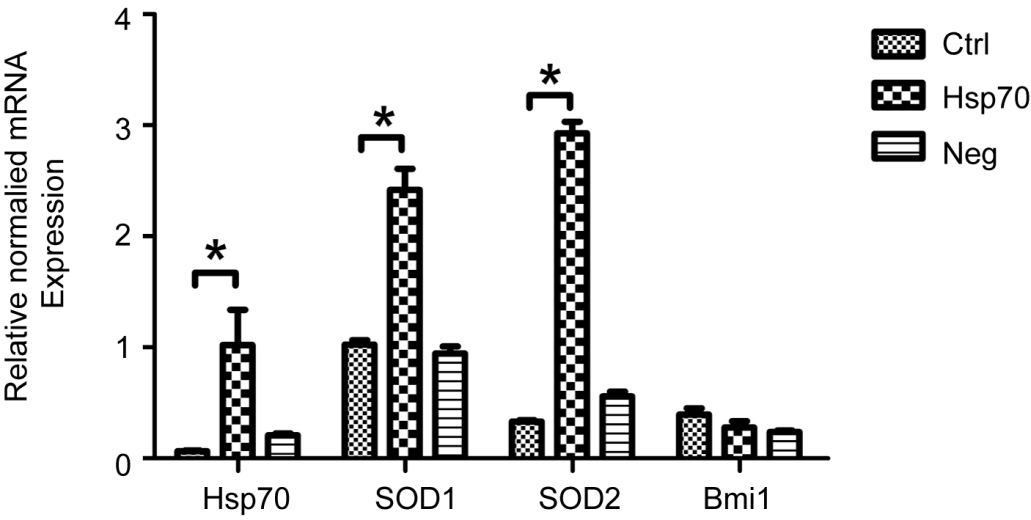


*Supplementary 2.* Supplementary Figure 2**: Hsp70, SOD1, SOD2 and Bmi1 mRNA Expression of SGNs *in vitro*.** Relative mRNA expressions of Hsp70, SOD1, SOD2 and Bmi1 were determined by quantitative RT-PCR. β-Actin RNA level was used as an endogenous control. Values are Means ± SD (n = 12 animals/group). **P* < 0.05 Hsp70. vs. control group.


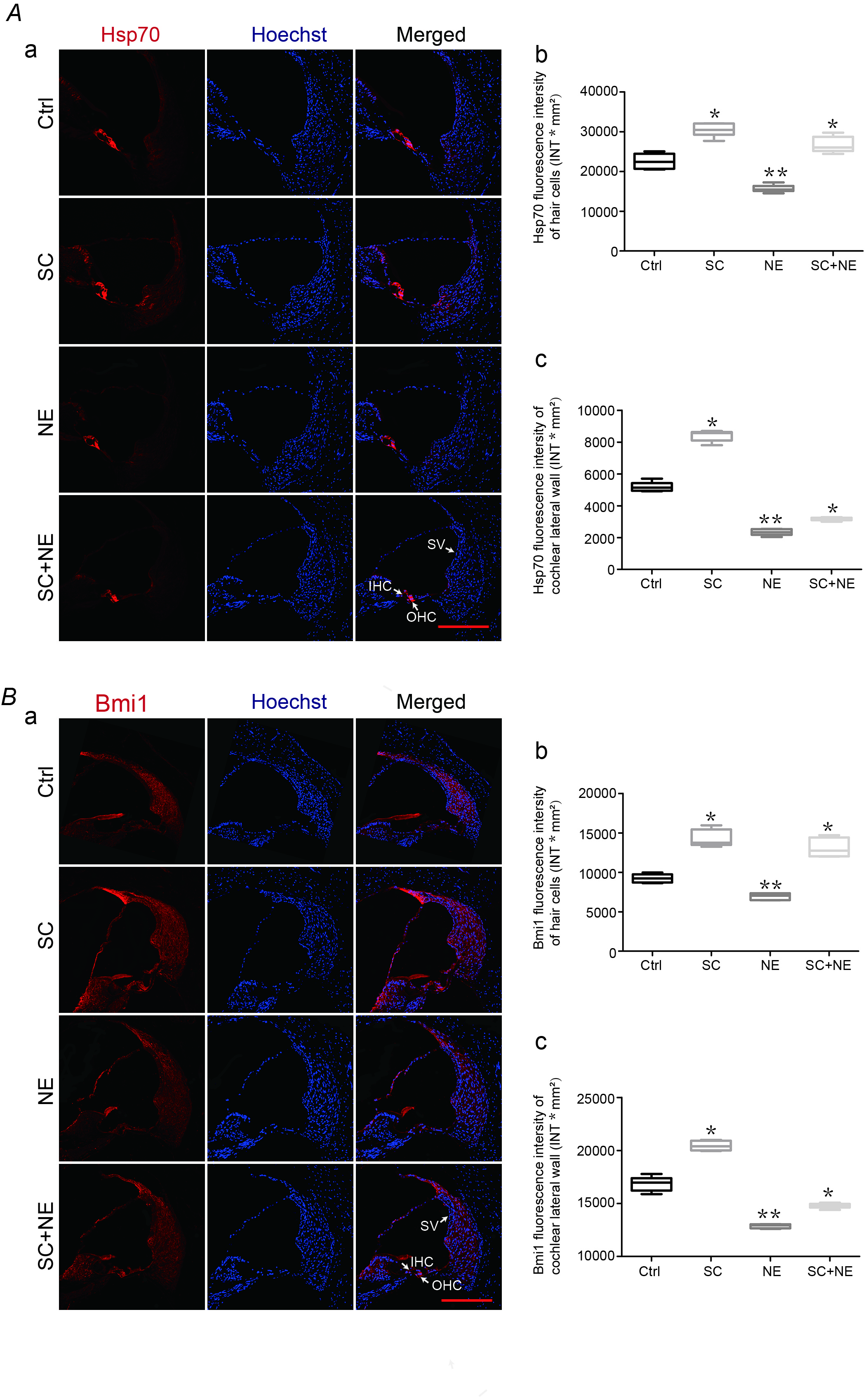


*Supplementary 3*. Supplementary Figure 3:**Hsp70, Bmi1 Protein Expressions in hair cells and cochlear lateral wall after Sound Conditioning and Acute Noise Exposure**. Distributions and expressions of Hsp70 **(A)**, Bmi1 **(B)** protein were detected with immunofluorescence assay in Ctrl and SC groups as well as NE and SC+NE groups **(a)**, while quantity of these Protein expressions in both hair cells **(b)** and cochlear lateral wall**(c)**was analyzed by fluorescence intensity. Hsp70/ Bmi1 (red) was labeled with fluorescent secondary antibody and nuclei (blue) was labeled with Hoechst. Scale bars represent 100µm. Furthermore, values are Means ± SD. Statistical analysis of the results presented in **(b)** (Hsp70: *F*=80.74, *P*<0.0001; Bmi1: *F*=86.64, *P*<0.0001;) and **(c)**(Hsp70: *F*=684.6, *P*<0.0001; Bmi1: *F*=338.2, *P*<0.0001;)was performed with one-way ANOVA, followed by Newman-Keuls’ post hoc test (**P*<0.05, ***P*<0.05; n=6 pictures from 3 animals/group).


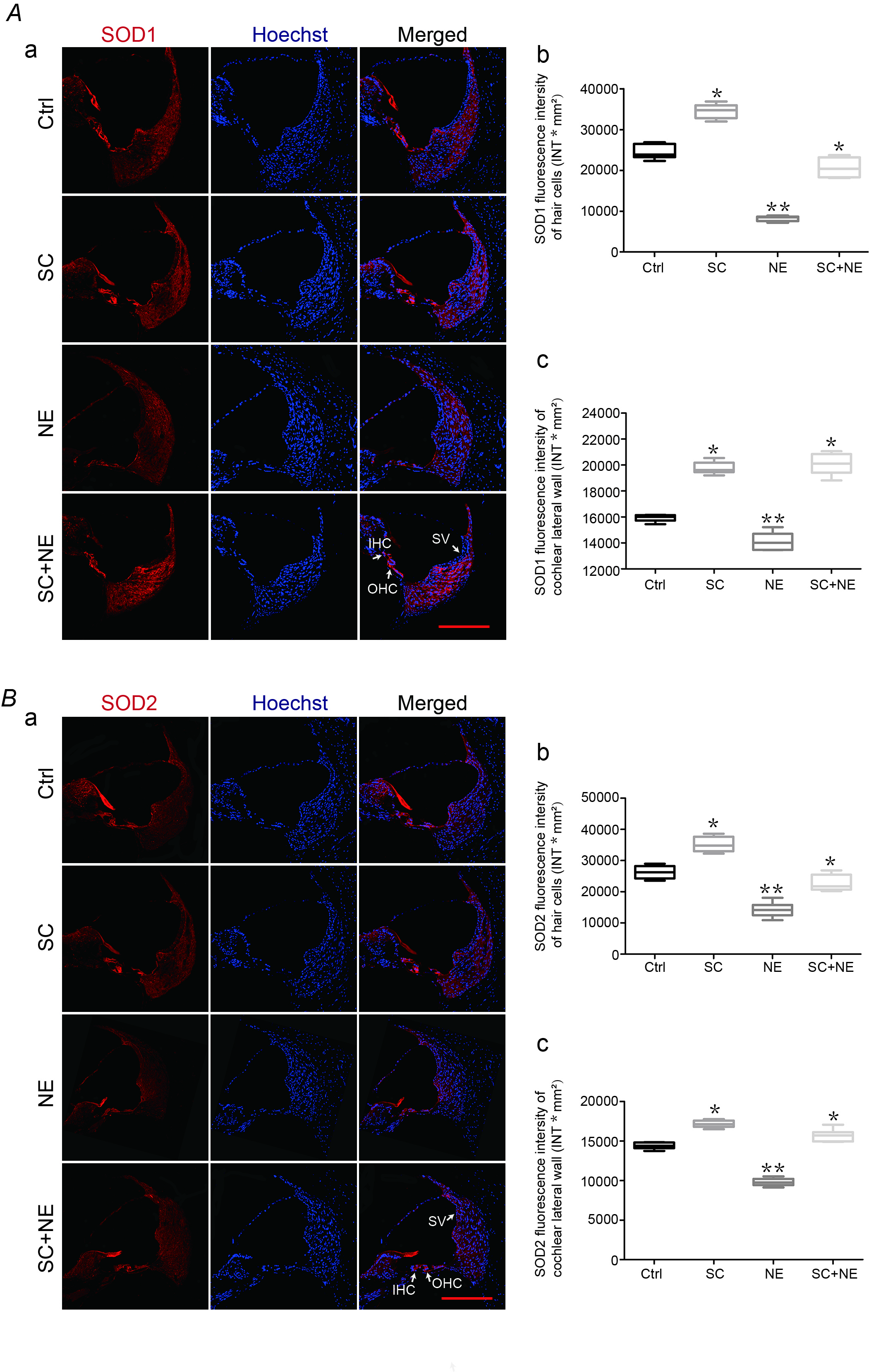


*Supplementary 4*. Supplementary Figure 4:**SOD1, SOD2 Protein Expressions in hair cells and cochlear lateral wall after Sound Conditioning and Acute Noise Exposure**. Distributions and expressions of SOD1**(A)**, SOD2**(B)** protein were detected with immunofluorescence assay in Ctrl and SC groups as well as NE and SC+NE groups **(a)**, while quantity of these Protein expressions in both hair cells **(b)** and cochlear lateral wall**(c)**was analyzed by fluorescence intensity. SOD1/ SOD2 (red) was labeled with fluorescent secondary antibody and nuclei (blue) was labeled with Hoechst. Scale bars represent 100µm. Furthermore, values are Means ± SD. Statistical analysis of the results presented in **(b)** (SOD1: *F*=233.0, *P*<0.0001; SOD2: *F*=79.93, *P*<0.0001;) and **(c)**(SOD1: *F*=143.0, *P*<0.0001; SOD2: *F*=199.8, *P*<0.0001;)was performed with one-way ANOVA, followed by Newman-Keuls’ post hoc test (**P*<0.05, ***P*<0.05; n=6 pictures from 3 animals/group).

| **Immunofluorescence assay of different groups in vivo and in vitro** | | | | | | | | | | |
| --- | --- | --- | --- | --- | --- | --- | --- | --- | --- | --- |
| **SGNs** | Hsp70 | | Bmi1 | | FoxO1 | | SOD1 | | SOD2 | |
| NE | *q*=25.66 | ***P*<0.05 | *q*=22.97 | ***P*<0.05 | *q*=21.19 | ***P*<0.05 | *q*=64.16 | ***P*<0.05 | *q*=54.47 | ***P*<0.05 |
| SC | *q*=10.87 | **P*<0.05 | *q*=50.33 | **P*<0.05 | *q*=08.14 | **P*<0.05 | *q*=12.14 | **P*<0.05 | *q*=14.70 | **P*<0.05 |
| **Hair cells** | Hsp70 | | Bmi1 | | SOD1 | | SOD2 | |  |  |
| NE | *q*=9.80 | ***P*<0.05 | *q*=6.30 | ***P*<0.05 | *q*=22.81 | ***P*<0.05 | *q*=12.40 | ***P*<0.05 |  |  |
| SC | *q*=11.18 | **P*<0.05 | *q*=13.78 | **P*<0.05 | *q*=14.12 | **P*<0.05 | *q*=9.20 | **P*<0.05 |  |  |
| **CLW** | Hsp70 | | Bmi1 | | SOD1 | | SOD2 | |  |  |
| NE | *q*=27.70 | ***P*<0.05 | *q*=22.62 | ***P*<0.05 | *q*=7.42 | ***P*<0.05 | *q*=20.44 | ***P*<0.05 |  |  |
| SC | *q*=31.10 | **P*<0.05 | *q*=20.32 | **P*<0.05 | *q*=15.66 | **P*<0.05 | *q*=12.25 | **P*<0.05 |  |  |
| **SGNs**  **in vitro** | Bmi1 | | FoxO1 | | SOD1 | | SOD2 | |  | |
| Hsp70 | *t*=15.55 | ***P*<0.05 | *t*=16.95 | ***P*<0.05 | *t*=4.82 | ***P*<0.05 | *t*=10.29 | ***P*<0.05 |  |  |
| PTC-209 | *t*=38.02 | **P*<0.05 | *t*=13.02 | **P*<0.05 | *t*=163.20 | **P*<0.05 | *t*=28.40 | **P*<0.05 |  |  |
|  |  |  |  |  |  |  |  |  |  |  |
|  |  |  |  |  |  |  |  |  |  |  |

*Supplementary 5*. Supplementary Table1:Statistical results of immunofluorescence of target proteins expression in vivo and in vitro(Student-Newman-Keuls (SNK)-*q* test for post-hoc comparisons (NE or SC vs. Ctrl) ***P*<0.05, **P*<0.05; Student’s *t*-test for the comparisons between the two groups (Hsp70 vs. Neg or PTC-209 vs. Ctrl)***P*<0.05, **P*<0.05.).

| **Western blot assay of different groups in vivo and in vitro** | | | | | | | | | | | |  |
| --- | --- | --- | --- | --- | --- | --- | --- | --- | --- | --- | --- | --- |
| **SGNs**  **in vivo** | Hsp70 | | | Bmi1 | | SOD1 | | SOD2 | |  | |  |
| NE | *q*=5.05 | | ***P*<0.05 | *q*=36.27 | ***P*<0.05 | *q*=45.12 | ***P*<0.05 | *q*=31.82 | ***P*<0.05 |  |  |  |
| SC | *q*=14.55 | **P*<0.05 | | *q*=16.01 | **P*<0.05 | *q*=10.26 | **P*<0.05 | *q*=5.57 | **P*<0.05 |  |  |  |
| **SGNs**  **in vitro** | Hsp70 | | | Bmi1 | | FoxO1 | | SOD1 | | SOD2 | |  |
| Hsp70 | *t*=66.27 | ***P*<0.05 | | *t*=22.48 | ***P*<0.05 | *t*=27.43 | ***P*<0.05 | *t*=14.65 | ***P*<0.05 | *t*=7.12 | ***P*<0.05 |  |
| PTC-209 | *t*=20.78 | **P*<0.05 | | *t*=61.81 | **P*<0.05 | *t*=50.89 | **P*<0.05 | *t*=50.72 | **P*<0.05 | *t*=37.38 | **P*<0.05 |  |
|  |  |  | |  |  |  |  |  |  |  |  |  |
|  |  |  | |  |  |  |  |  |  |  |  |  |

*Supplementary 6*. Supplementary Table 2:Statistical results of Western blot of target proteins expression in vivo and in vitro(Student-Newman-Keuls (SNK)-*q* test for post-hoc comparisons (NE or SC vs. Ctrl) ***P*<0.05, **P*<0.05; Student’s *t*-test for the comparisons between the two groups (Hsp70 vs. Neg or PTC-209 vs. Ctrl)***P*<0.05, **P*<0.05.).
